# Olfactomedin 4 mediation of prostate stem/progenitor-like cell proliferation and differentiation via MYC

**DOI:** 10.1038/s41598-020-78774-5

**Published:** 2020-12-14

**Authors:** Hongzhen Li, Vijender Chaitankar, Jianqiong Zhu, Kyung Chin, Wenli Liu, Mehdi Pirooznia, Griffin P. Rodgers

**Affiliations:** 1 Molecular and Clinical Hematology Branch, National Heart, Lung, and Blood Institute, National Institutes of Health, Bldg. 10, Room 9N119, 9000 Rockville Pike, Bethesda, MD 20892 USA; 2 Bioinformatics and Systems Biology Core, National Heart, Lung, and Blood Institute, National Institutes of Health, Bethesda, MD 20892 USA

**Keywords:** Cell biology, Stem cells

## Abstract

Olfactomedin 4 (*OLFM4*) is expressed in normal prostate epithelial cells and immortalized normal human prostate epithelial cells (RWPE1), but the identity of *OLFM4*-expressing cells within these populations and OLFM4’s physiological functions in these cells have not been elucidated. Using single-cell RNA sequencing analysis, we found here that OLFM4 was expressed in multiple stem/progenitor-like cell populations in both the normal prostate epithelium and RWPE1 cells and was frequently co-expressed with KRT13 and LY6D in RWPE1 cells. Functionally, *OLFM4*-knockout RWPE1 cells exhibited enhanced proliferation of the stem/progenitor-like cell population, shifts stem/progenitor-like cell division to favor symmetric division and differentiated into higher levels PSA expression cells in organoid assays compared with *OLFM4*-wild RWPE1 cells. Bulk-cell RNA sequencing analysis pinpointed that *cMYC* expression were enhanced in the *OLFM4*-knockout RWPE1 cells compared with OLFM4-wild cells. Molecular and signaling pathway studies revealed an increase in the WNT/APC/MYC signaling pathway gene signature, as well as that of *MYC* target genes that regulate multiple biological processes, in *OLFM4*-knockout RWPE1 cells. These findings indicated that OLFM4 is co-expressed with multiple stem/progenitor cell marker genes in prostate epithelial cells and acts as a novel mediator in prostate stem/progenitor cell proliferation and differentiation.

## Introduction

Prostate stem/progenitor cells exist within prostatic pseudostratified epithelium and exhibit the capacities of self-renewal and multi-lineage differentiation that underly prostate organogenesis and homeostasis. The stem cells self-renew via one stem cell giving rise to two stem cells (symmetric cell division) and differentiate via one stem cell giving rise to one stem-cell copy and one progenitor cell (asymmetric cell division), and the one progenitor cell subsequently giving rise to two differentiated cells (committed cell division)^1–3^. Studies of prostate stem/progenitor cells have been performed using cell-surface markers, such as CD133, CD44, CD49F, CD24, and CD26, that are found on enriched putative stem/progenitor cells in human and mouse normal or tumor prostate tissues^4–6^. Recently, cytokeratin (CK) 13 (KRT13) has been identified as a marker for normal human prostate epithelial stem/progenitor cells, as well as bone marrow metastatic cancer cells^7–9^. The cytokeratins and other specific molecular markers have been used as epithelial cell markers for identifying cell types in the prostate epithelium. For example, CK5, CK8, CK18, and CK 19 are intermediate cell markers, CK5, CK14, and p63 are basal cell markers, and CK8, CK18, and androgen receptor (AR) are luminal cell markers; synaptophysin serves as a rare neuroendocrine cell marker in the developing and adult prostate epithelium^10–13^.

Experimental studies of properties of stem/progenitor cells have been performed using prostate regeneration, tissue recombination, and genetic marker tracing in rodent models^14–16^. Studies of human prostate epithelial stem/progenitor cells have been performed using primary two-dimensional (2D) cell culture, Matrigel three-dimensional (3D) cell culture, and combination xenograft ex vivo models^12,17,18^. Recently, single-cell RNA sequencing technology has been performed for identifying putative stem/progenitor cells in normal adult prostate epithelium and in primary-culture human benign prostate epithelial cells^8,9^. We have also previously identified putative prostate stem/progenitor cells in immortalized human normal and benign prostate epithelial cell populations^19,20^. The RWPE1 immortalized human normal prostate epithelial cell line^21^ that we used in those studies has been widely used as a benign prostate epithelial cell line for molecular, cellular, and biological studies.

The WNT/APC/MYC signaling pathways play an important role in prostate development and prostate-cancer progression by mediating prostate stem/progenitor cell functions^22,23^. MYC protein serves as a master regulator of cell proliferation, metabolism, ribosome biogenesis, protein synthesis, and mitochondrial function^24^. MYC protein targets most cell-cycle regulator genes, including cyclins, cyclin-dependent kinases, and cell-cycle inhibitors, drives quiescent cells to enter the cell cycle, and promotes cell proliferation^24,25^. The *MYC* oncogene is somatically amplified in a subset of advanced prostate cancer^26^. Overexpression of MYC mRNA has been found in both prostate intraepithelial neoplasia and carcinomas^27,28^, and overexpression of MYC protein has been reported as an early alteration in human prostate carcinogenesis^29^. The *Myc*-driven murine prostate-cancer model mimics the progression of human prostate cancer, in which *MYC* is overexpressed^30,31^.

The olfactomedin 4 (*OLFM4*) gene was first cloned from human myeloid progenitor cells and is normally expressed in prostate, bone marrow, small intestine, and pancreas^32^. The *OLFM4* gene plays an important role in innate immunity, inflammation, and cancers^33^. We have reported previously that *OLFM4* gene expression was reduced or lost during the progression of prostate cancer due to frequent genetic deletion^34^ and hypermethylation of the *OLFM4* gene promoter region^35^. The expression of OLFM4 mRNA has been detected in normal prostate tissues, primary-cultured normal prostate epithelial cells, and RWPE1 immortalized normal human prostate epithelial cells^34–36^. However, *OLFM4*-expressing cell subtypes and OLFM4’s physiological functions in those cells remain elusive.

In this study, we sought to identify and characterize the *OLFM4*-expressing cells found within prostate epithelial cells. We identified *OLFM4*-expressing cells as belonging to multiple stem/progenitor cell populations in both the normal prostate epithelium and in RWPE1 cells. We demonstrated that knockout of the *OLFM4* gene in RWPE1 cells enhanced the proliferation of stem/progenitor-like cell populations, shifts stem/progenitor-like cell division to favor symmetric division, and differentiated into higher levels PSA expression cells in organoid assays when compared with *OLFM4*-wild RWPE1 cells. Furthermore, gene set enrichment analysis of RNA-sequencing results for RWPE1 stem/progenitor-like cells revealed that *OLFM4* knockout enriched gene signatures related to stem cells, which were subsequently identified to be related to the WNT/APC/MYC signaling pathway. Taken together, our results suggest that *OLFM4*-expressing prostate epithelial cells represent multiple stem/progenitor cell populations and that OLFM4 mediates those cells’ proliferation and differentiation through the WNT/APC/MYC signaling pathway.

## Results

### *OLFM4* is highly co-expressed with stem/progenitor cell markers in normal human adult prostate epithelium

**Figure 1:**
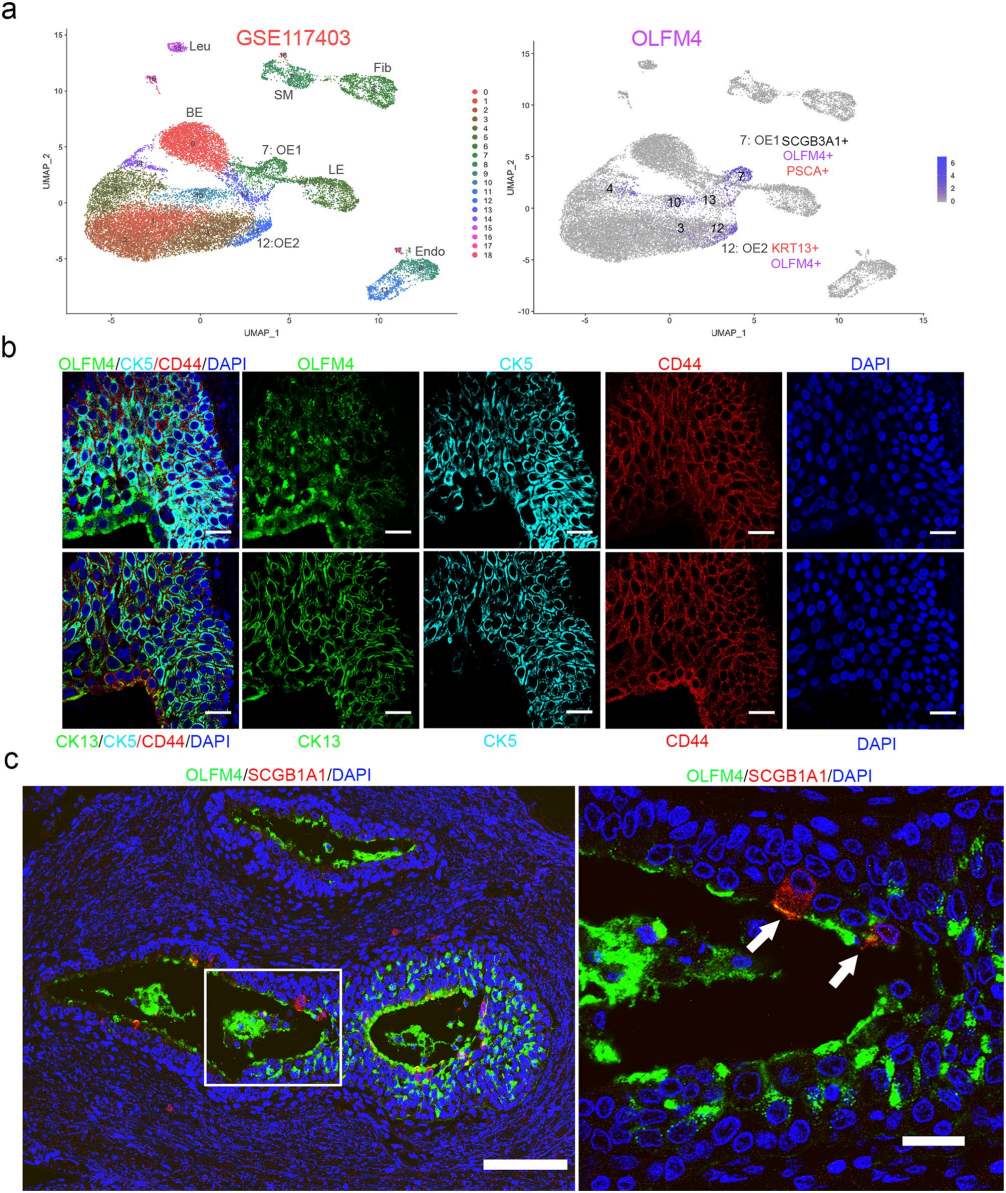
Identification of *OLFM4*-expressing cells in normal human adult prostate epithelium. (**a**) Uniform Manifold Approximation and Projection (UMAP) plots of integrated data from single-cell RNA sequencing of the GSE117403 dataset. Left panel shows 19 clusters of total prostate cells; right panel shows OLFM4-expressing cells (purple color) in clusters 7 and 12. (**b**) Representative triple-color immunofluorescent staining of normal prostatic urethra tubular epithelium. OLFM4 and CK13 (green); CD44 (red); CK5 (cyan); DAPI (blue). Scale bar: 20 µm. (**c**) Representative double-color immunofluorescent staining of normal prostatic tissues. OLFM4 (green); SCGB1A1 (red); DAPI (blue). Left panel shows low-magnification image. Scale bar: 100 µm. Right panel shows high-magnification image. Scale bar: 20 µm. Arrow indicates OLFM4+/SCGB1A1+ cells.

To identify *OLFM4*-expressing cells in the normal human prostate, we downloaded a dataset from single-cell RNA sequencing for normal human prostate (GSE117403, which is a part of super series GSE120716^8^) and analyzed cells for gene-expression signatures using Uniform Manifold Approximation and Projection (UMAP) software. Analysis of differentially expressed genes confirmed 4 epithelial clusters and 4 stromal clusters (leukocyte, endothelium, fibroblast, and smooth muscle) within 19 clusters (Fig. 1a, left panel)^8^. Higher populations of OLFM4-expressing cells were confirmed in clusters 7 and 12 and lower populations were identified in four other epithelial clusters (Fig. 1a, right panel and Supplementary Table S1). OLFM4 was highly co-expressed with SCGB3A1, LCN2, PIGR, PSCA (prostate stem cell antigen), and CD24, which are all prostate stem-cell markers, in cluster 7, and with KRT13, APOBEC3A, LYPD3, and KRT19, which are all prostate stem/progenitor cell markers, in cluster 12 (Fig. 1a, right panel, Supplementary Fig. S1, and Table S1). Cluster 7 has previously been reported to belong to Club cells, which express SCGB1A1, and cluster 12 has previously been reported to belong to Hillock epithelial cells, which express higher levels of KRT13 markers in the human normal prostate epithelia^8^ and in the mouse lung epithelia^37^.

We further classified OLFM4-expressing prostate epithelial cells by using markers for stem/progenitor cells (KRT13 [CK13] for cluster 12 cells, SCGB1A1 for cluster 7 cells and CD44+ cells); basal cells (CK5, CK14); and luminal cells (CK8). Triple-color immunofluorescent staining with specific antibodies demonstrated the expression of OLFM4 in the CK13+, CD44+, and CK5+ cells, where multiple layers of epithelia are found in the prostatic urethra tube (Fig. 1b). We observed about 6% (12/187) cells are expressing SCGB1A1 among OLFM4 positive cells in the prostatic urethra tube and peri-urethra tube epithelia (Fig. 1c). OLFM4+/CK8+ cells were found within multiple layers of epithelia in the prostatic urethra and peri-urethra tube and a few in the distal regions of the prostatic gland (Supplementary Fig. S2). Further, OLFM4+/CK8+/FOXA1+ cells were observed in the distal regions of prostatic gland (Supplementary Fig. S2) and prostatic acini (Supplementary Fig. S2). Double-color immunofluorescent staining showed that OLFM4+/CD44+ cells are distributed within CD44+ epithelial cells in the prostate acini (Supplementary Fig. S2). OLFM4+/CK14− and OLFM4+/CK8− cells are present within epithelial cells in the prostate acini (Supplementary Fig. S2). Taken together, these results suggest that OLFM4-expressing cells are CK13+/CD44+, SCGB1A1+ stem/progenitor cells and CK8+ luminal progenitor cells.

### *OLFM4* is highly co-expressed with stem/progenitor cell markers in RWPE1 cells

**Figure 2:**
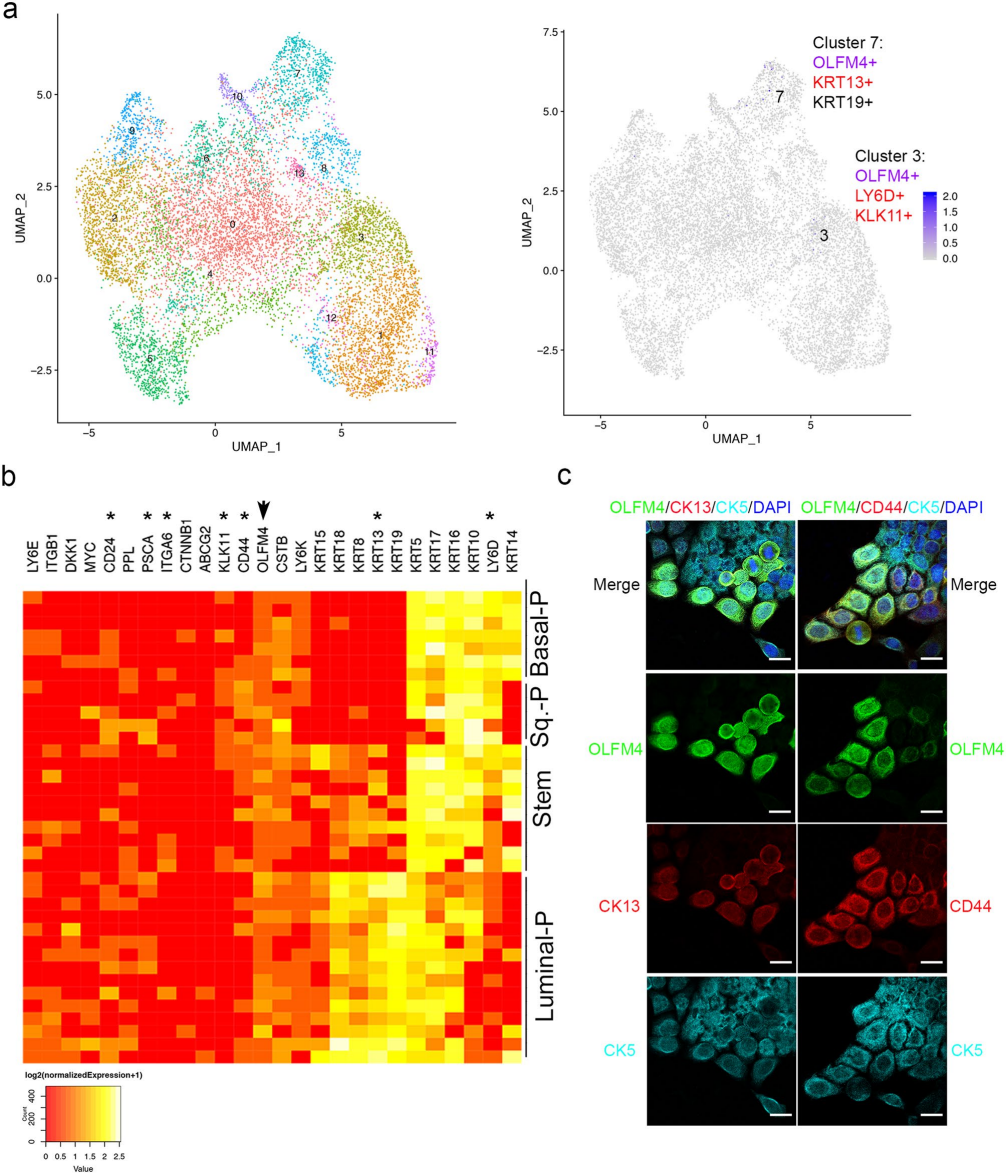
Identification of *OLFM4*-expressing RWPE1 cells. (**a**) Uniform Manifold Approximation and Projection (UMAP) plots of integrated data from single-cell RNA sequencing of RWPE1 cells. Left panel shows 13 clusters of total RWPE1 cells; right panel shows OLFM4-expressing cells (purple color) in clusters 3 and 7. (**b**) Heat map illustrates OLFM4 co-expression with stem/progenitor-cell marker genes, cytokeratins, and others in 37 OLFM4-expressing RWPE1 cells. Sq., squamous. P, progenitor. *Indicates lines for stem/progenitor-cell marker genes; arrow indicates line for the *OLFM4* gene. (**c**) Representative triple-color immunofluorescent staining of RWPE1 cells. OLFM4 (green); CK13 and CD44 (red); CK5 (cyan); DAPI (blue). Scale bar: 20 µm.

We have previously detected OLFM4 RNA and protein expression in RWPE1 cells^35^. RWPE1 cells are immortalized normal adult prostate epithelial cells whose growth can be maintained under serum-free conditions in 2D culture. We sought to identify OLFM4-expressing cells in the RWPE1 cell population through single-cell RNA sequencing of a total of 5000 single cells obtained from 2D culture. Thirteen clusters were identified by analyzing gene-expression signatures with Uniform Manifold Approximation and Projection (UMAP) software (Fig. 2a, left panel). High numbers of OLFM4-expressing cells were located in cluster 7, in which the stem/progenitor genes KRT13 and KRT19 were also expressed, and in cluster 3, in which the stem/progenitor genes LY6D and KLK11 were also expressed (Fig. 2a, right panel and Supplementary Fig. S3). The higher level of OLFM4-expressing cells distributed in the stem/progenitor-like cell populations was shown in a heat map generated from single-cell RNA sequencing of RWPE1 cells (Fig. 2b). We detected a 0.74% OLFM4 RNA expression rate (that is, OLFM4 expression was observed in 37 cells from the total of 5000 single RWPE1 cells that were RNA sequenced). As shown in the heat map, the population of OLFM4-expressing cells that were stem-like cells was 27.0% (10 out of 37), that were basal progenitor-like cells was 18.9% (7 out of 37), that were luminal progenitor-like cells was 40.5% (15 out of 37), and that were squamous progenitor-like cells was 13.5% (5 out of 37). Several cells expressed different combinations of stem/progenitor-cell marker genes, such as *KRT13*, *LY6D*,* PSCA*,* CD44*,* ITGA6*, and *CD24*. Further analysis of stem/progenitor cells found that 78.4% of OLFM4+ cells were LY6D+ (29 out of 37), 54.1% of OLFM4+ cells were KRT13+ (20 out of 37), 45.9% of OLFM4+ cells were CD44+ (17 out of 37), 43.2% of OLFM4+ cells were CD24+ (16 out of 37), 18.9% of OLFM4+ cells were PSCA+ (7 out of 37), and 18.9% of OLFM4+ cells were ITGA6+ (7 out of 37). The results suggest that multiple stem/progenitor cell populations exist in RWPE1 cells and OLFM4 was more frequently co-expressed with LY6D and KRT13 stem-cell markers.

Examination of RWPE1 cells with triple-color immunofluorescent staining demonstrated that OLFM4 was co-expressed with CK13, CD44, CK5 and SCGB1A1 (Fig. 2c, Supplementary Fig. S3). We further observed that OLFM4-positive cells co-expressed with CK8 cell markers (Supplementary Fig. S3). OLFM4-positive cells did not express P63, AR, and synaptophysin markers (Supplementary Fig. S3). These results verified single-cell RNA sequencing data indicating that OLFM4 is expressed in multiple stem/progenitor-like cell populations in RWPE1 cells.

### *OLFM4*-knockout RWPE1 cells are enriched in CD49F+ and CD44+ stem/progenitor-like cell populations

**Figure 3:**
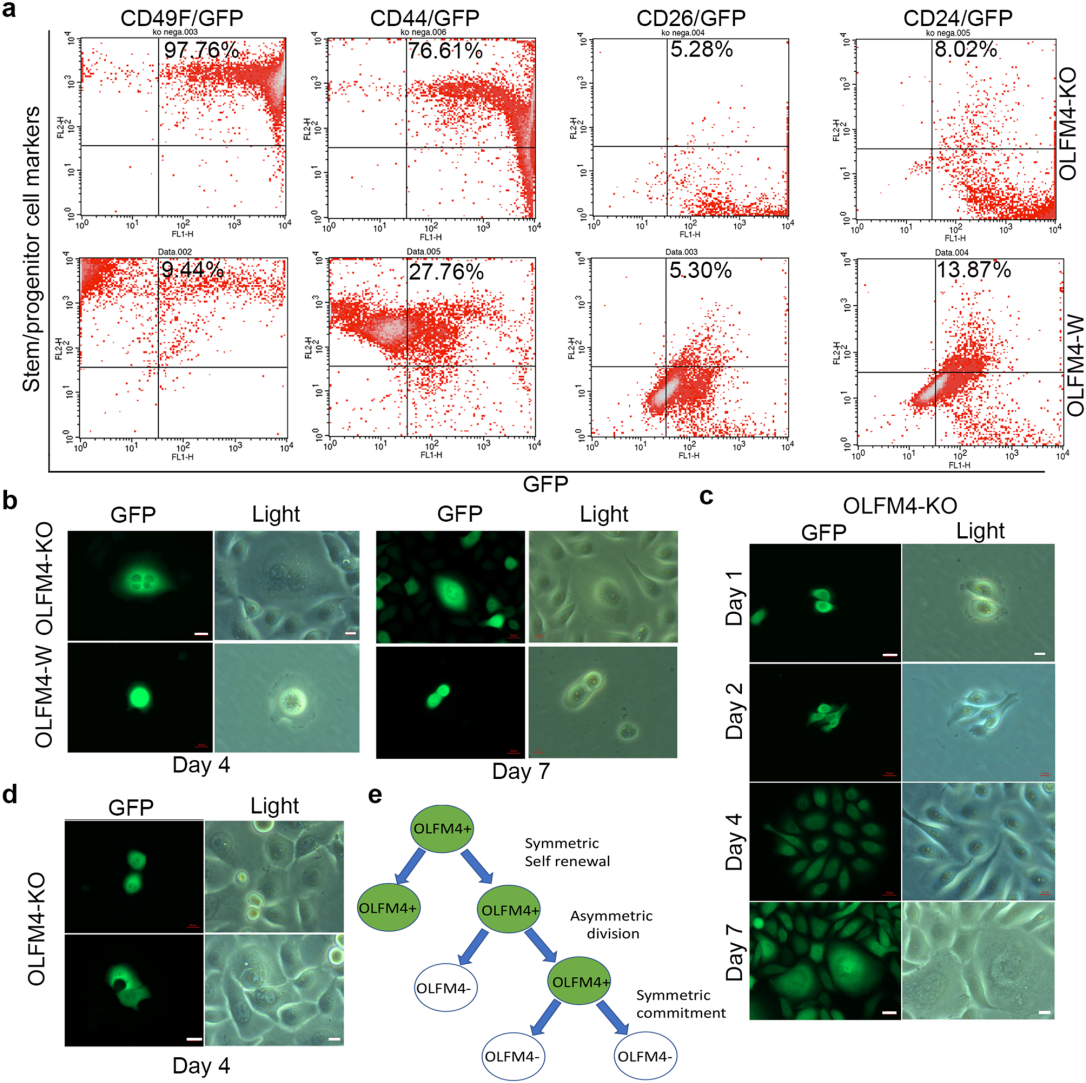
Characterization of *OLFM4*-knockout GFP reporter RWPE1 cells in 2D culture. (**a**) Representative FACS analysis of *OLFM4*-wild and *OLFM4*-knockout GFP reporter RWPE1 cells using antibodies to GFP combined with antibodies to stem/progenitor-cell markers CD49F, CD44, CD26, or CD24. Inset values indicate the percentage of double-marker—positive cell populations. (**b**) Representative GFP- and light-field images generated using single-cell tracing of *OLFM4*-wild and *OLFM4*-knockout GFP reporter RWPE1 cells at 4 and 7 days in 2D culture. Scale bar: 10 µm. (**c**) Representative GFP- and light-field images of *OLFM4*-knockout GFP reporter RWPE1 cells at 1, 2, 4, and 7 days in 2D culture. Scale bar: 10 µm. (**d**) Representative GFP- and light-field images of *OLFM4*-knockout GFP reporter RWPE1 cells at 4 days in 2D culture. Scale bar: 10 µm. (**e**) Illustration of *OLFM4*-expressing GFP-positive RWPE1 stem/progenitor-like cell symmetric and asymmetric division. OLFM4-KO, knockout; OLFM4-W, wild.

Our earlier data demonstrated that OLFM4 was expressed in multiple stem/progenitor cell types within the wild-type OLFM4 RWPE1 cell population. To study *OLFM4* gene function in human prostate stem/progenitor-like cells, we used CRISPR/Cas9 technology to establish *OLFM4*-knockout and *OLFM4*-wild RWPE1 cell clones that express green fluorescent protein (GFP). The cell clones were verified with genomic PCR sequencing and RT-PCR (Supplementary Fig. S4). We then performed FACS analysis using anti-GFP antibody combined with phycoerythrin (PE)-labeled antibodies to the cell-surface stem/progenitor-cell markers CD49F, CD44, CD26, or CD24. GFP+/CD49F+ cells and GFP+/CD44+ cells were highly enriched in *OLFM4*-knockout GFP reporter RWPE1 cells compared with *OLFM4*-wild GFP reporter RWPE1 cells (Fig. 3a, left two columns of panels). In contrast, GFP+/CD26+ cells and GFP+/CD24+ cells were not enriched in *OLFM4*-knockout GFP reporter RWPE1 cells compared with *OLFM4*-wild GFP reporter RWPE1 cells (Fig. 3a, right two columns of panels). These results indicate that *OLFM4* knockout enriched CD49F+ and CD44+ cell populations in RWPE1 cells.

### *OLFM4*-knockout RWPE1 stem/progenitor-like cells exhibit enhanced growth via symmetric division in 2D culture

To identify individual RWPE1 stem/progenitor-like cells, we performed time course single-GFP+ cell tracing on 2D cultures of *OLFM4*-wild and *OLFM4*-knockout GFP reporter RWPE1 cells that were generated by transfecting the CRISPR/Cas 9 OLFM4 activation or knock out plasmids into the RWPE1 *OLFM4*-wild cells (Supplementary Fig. S4). We found that *OLFM4-*knockout stem/progenitor-like cells proliferated by undergoing both symmetric division (self-renewal) and asymmetric division (giving rise to one stem cell and one progenitor cell) at day 4 to day 7 (Fig. 3b,c). We also found symmetric commitment *OLFM4*-knockout GFP reporter RWPE1 cells (Fig. 3d). In contrast, we observed that *OLFM4*-wild GFP reporter RWPE1 cells stayed in quiescence and slowly asymmetric divided at day 4 to day 7 (Fig. 3b and Supplementary Fig. S4). To quantify the number of symmetric and asymmetric cells produced by each GFP reporter cell line, the GFP-positive cells at day 4 of 2D culture were counted. At that timepoint, 20.1% (44/219) of *OLFM4*-wild GFP reporter RWPE1 cells were in symmetric division and 79.9% (175/219) were in asymmetric division (Fig. 3e). In contrast, 70.6% (204/289) of *OLFM4*-knockout GFP reporter RWPE1 cells were in symmetric division and 29.4% (85/289) were in asymmetric division. These results suggest that *OLFM4* promotes stem/progenitor-like cell asymmetric division, whereas *OLFM4* knockout shifts stem/progenitor-like cell division to favor symmetric division.

### *OLFM4*-GFP reporter RWPE1 stem/progenitor-like cells proliferate and differentiate in 3D Matrigel culture

**Figure 4:**
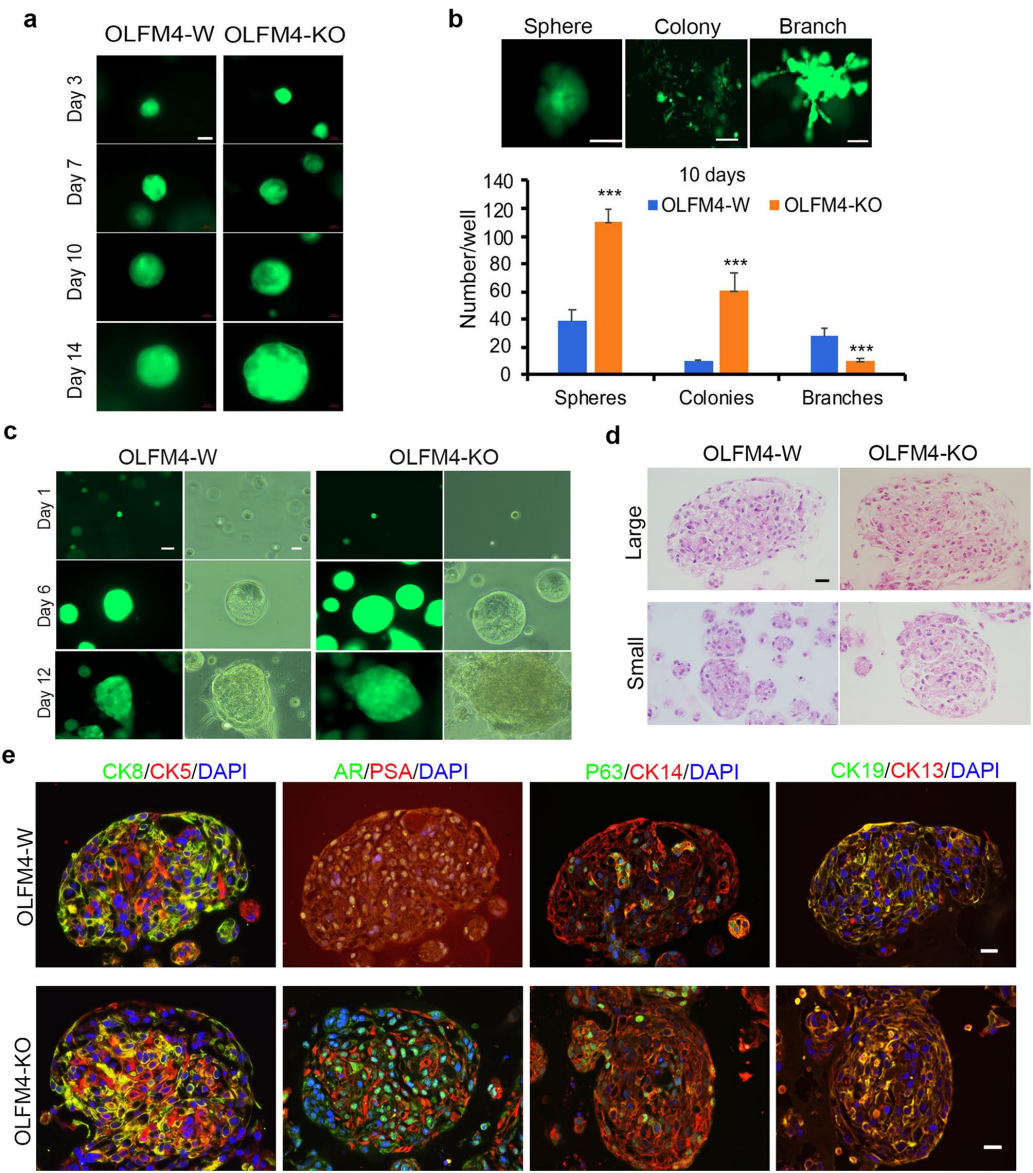
Characterization of *OLFM4*-GFP reporter RWPE1 cells in 3D Matrigel culture. Prostate sphere-formation and organoids assays were performed with *OLFM4*-wild and *OLFM4*-knockout GFP reporter RWPE1 cells grown in Matrigel. (**a**) Representative GFP-field sphere images using single-cell tracing over time (scale bar: 20 µm). (**b**) Representative GFP-field images of sphere, colony and branch formation after 10 days in Matrigel culture. Scale bar: 100 µm. Bar graph presents mean number (± standard deviation; SD, n = 6) of spheres (> 50 µm in diameter), colonies, or branches formed after 10 days in culture. ***p < 0.001 when compared with *OLFM4*-wild RWPE1 cells (Student’s t-test). (**c**) Representative GFP- and light-field images generated using single-cell tracing of *OLFM4*-wild (W) and *OLFM4*-knockout (KO) GFP reporter RWPE1 cells grown to organoids at 1, 6, and 12 days in culture. Scale bar: 20 µm (GFP) or 10 µm (light). (**d**) Representative hematoxylin–eosin staining of organoids at 12 days in culture. Scale bar: 20 µm. (**e**) Representative double-color immunofluorescent staining of large organoids at 12 days in culture. DAPI (blue) was used for nuclei staining. Scale bars: 20 µm. OLFM4-W, OLFM4-wild; OLFM4-KO, OLFM4-knockout.

Matrigel 3D sphere formation assays and organoid assays has previously been used to evaluate the self-renewal and differentiation potential of stem/progenitor cells^19,38,39^. To identify properties of *OLFM4*-expressing RWPE1 stem/progenitor-like cells, *OLFM4*-knockout or *OLFM4*-wild GFP reporter RWPE1 cells were analyzed in 3D Matrigel culture using prostate sphere-formation assays and organoid assays from single-GFP+ cells. We observed single-GFP+ cells to follow sphere formation from day 1 to day 14 under sphere culture conditions (Fig. 4a). We observed three growth patterns: spheres within the Matrigel; colonies attached to the surface of plates; and branches within the Matrigel in the sphere formation assays (Fig. 4b). *OLFM4*-knockout GFP reporter RWPE1 cells formed significantly more spheres and colonies but fewer branches than *OLFM4*-wild GFP reporter RWPE1 cells (Fig. 4b). The cell populations found within the spheres were identified by immunohistochemical staining (Supplementary Fig. S5). All *OLFM4*-knockout RWPE1 sphere formed cells expressing CD44 stem/progenitor cell marker and CK5/CK14 basal cell markers. Most of cells expressing epithelial marker, E-cadherin and some cells expressing

mesenchymal cell marker, vimentin. We did not detect differentiated luminal cell markers CK8 and AR as well as epithelial-mesenchymal transition marker, N-cadherin expression in those cells (Supplementary Fig. S5).

To further identify differentiation abilities of *OLFM4*-expressing RWPE1 stem/progenitor-like cells, we performed organoid culture assays with *OLFM4*-wild and *OLFM4*-knockout GFP reporter RWPE1 cells. We traced single cells using the GFP reporter protein to follow organoid formation from day 1 to day 12 under organoid culture conditions (Fig. 4c). Both large and small organoids with compact cells exhibiting basal stem/progenitor cell-like morphology were observed after 12 days of culture (Fig. 4d)^17^. We performed double-color immunofluorescent staining of organoids after 12 days of culture for identifying cell populations within the large and small organoids. We detected the expression of luminal cell markers (CK8, AR), basal cell markers (CK5, CK14, and P63), and stem/progenitor cell markers (CK19 and CK13) in the large organoids obtained from both *OLFM4*-wild and *OLFM4*-knockout GFP reporter RWPE1 cells (Fig. 4e). Interestingly, we observed a few cells presented lower level PSA expression in the large organoids from *OLFM4*-wild GFP reporter RWPE1 cells. In contrast, we observed more than half cells presented higher level PSA expression in the large and small organoids from *OLFM4*-knock out GFP reporter RWPE1 cells (Fig. 4e and Supplementary Fig. S5).

Taken together, these data suggest that *OLFM4*-knockout RWPE1 cells exhibit higher proliferative abilities and differentiated into higher levels PSA expression cells when compared with *OLFM4*-wild RWPE1 cells.

### *OLFM4*-knockout RWPE1 cells exhibit enhanced MYC-signaling target gene signatures

**Figure 5:**
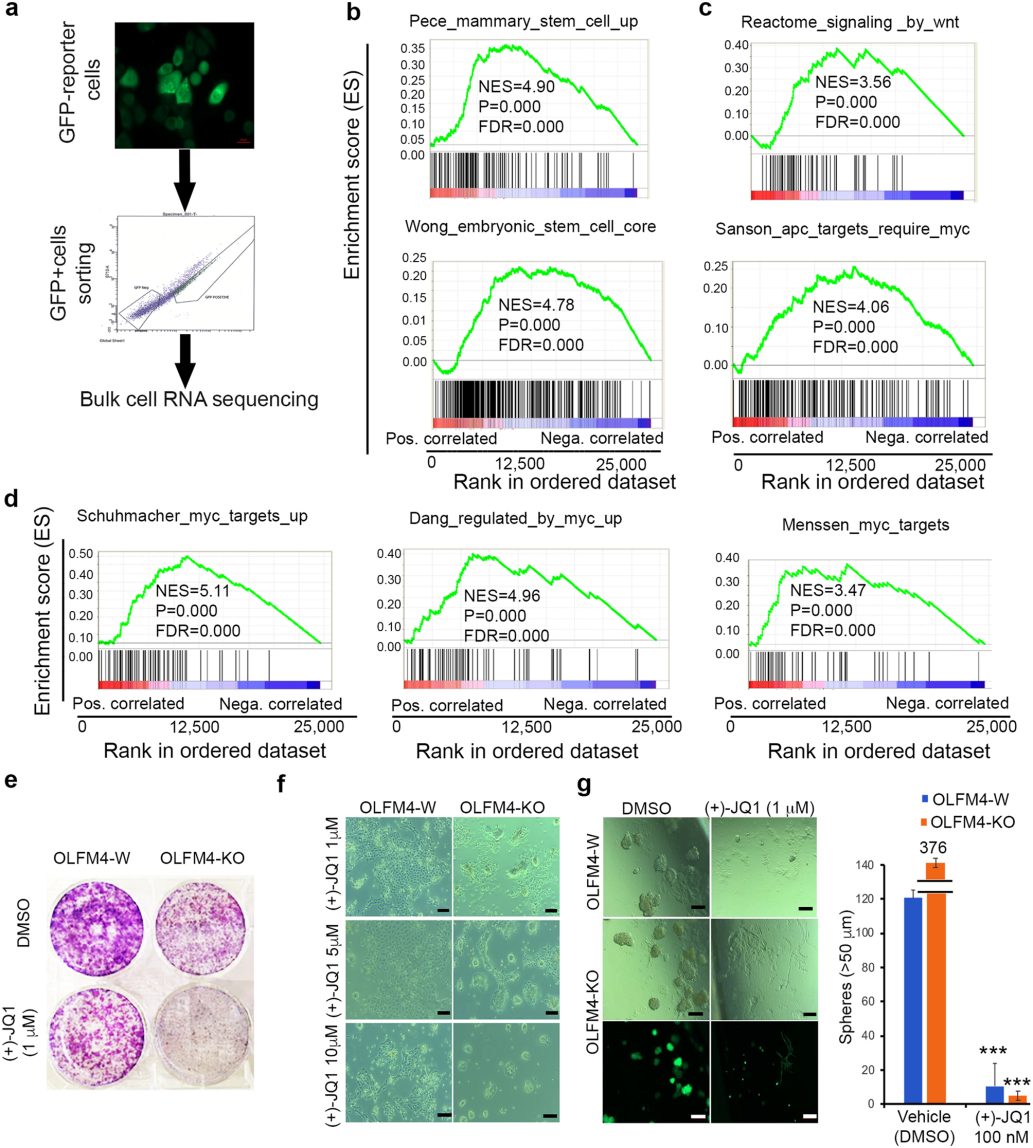
GSEA analysis for *OLFM4*-knockout GFP reporter RWPE1 cells from bulk-cell RNA sequencing data. (**a**) Strategy of bulk-cell RNA sequencing for FACS cell sorting by GFP-marker expression of *OLFM4*-GFP reporter RWPE1 cells. (**b**) GSEA showing enrichment of stem-cell-like gene signatures in *OLFM4*-knockout GFP reporter RWPE1 cells. (**c**) GSEA showing enrichment of *WNT*-signaling and *APC*/*MYC*-signaling target gene signatures in *OLFM4*-knockout GFP reporter RWPE1 cells. (**d**) GSEA showing enrichment of MYC target gene signatures in *OLFM4*-knockout GFP reporter RWPE1 cells from three different data resources. (**e**) Representative images of colonies of *OLFM4*-wild and *OLFM4*-knockout GFP reporter RWPE1 cells cultured in the presence of 1 µM (+)-JQ1 (or DMSO, vehicle control) for 7 days in 6-well plates, then stained with the Diff-Quik Stain Set. (**f**) Representative images of colonies of *OLFM4*-wild and *OLFM4*-knockout GFP reporter RWPE1 cells cultured in the presence of 1 µM, 5 µM, or 10 µM (+)-JQ1 (or DMSO, vehicle control) for 7 days. Scale bar: 100 µm. (**g**) Representative GFP- and light-field images of spheres of *OLFM4*-wild and *OLFM4*-knockout GFP reporter RWPE1 cells cultured in the presence of 1 µM (+)-JQ1 (or DMSO, vehicle control) for 10 days in 3D culture. Scale bar: 100 µm. Bar graph presents mean number of spheres (± SD, n = 6) from these experiments using 100 nM of (+)-JQ1 (or DMSO, vehicle control). W, wild; KO, knockout. ***p < 0.001 (Student’s t-test).

To explore the molecular mechanisms underlying OLFM4 mediation of RWPE1 cell self-renewal and differentiation, we performed bulk-cell RNA sequencing analysis (Fig. 5). *OLFM4*-knockout RWPE1 cells were sorted by GFP-marker expression, then bulk-cell RNA sequencings were performed (Fig. 5a). We first analyzed and performed quantitative real-time RT-PCR for picked genes. We found expression of stem-cell marker genes *KRT13*, *LY6D*, *KLK10*, and *ITGA6* were enhanced, but the luminal progenitor cell marker genes *CD24* and *PSCA* were reduced in *OLFM4*-knockout GFP reporter RWPE1 cells compared with *OLFM4*-wild RWPE1 cells (Supplementary Fig. S6). The *MYC* gene was significantly increased, while in contrast other transcription factors, such as prostate specific transcription factor, *HOXB13*, *NKX3.1*, and *BMI1*, were reduced in *OLFM4*-knockout GFP reporter RWPE1 cells compared with *OLFM4*-wild RWPE1 cells (Supplementary Fig. S6). The RNA expression of basal cell marker genes, *KRT5* and *KRT14*, were increased (Supplementary Fig. S6) but luminal cell marker genes *KRT8* and *KRT18* were reduced (Supplementary Fig. S6) in *OLFM4*-knockout GFP reporter RWPE1 cells compared with *OLFM4*-wild RWPE1 cells. These results were consistent with FACS data that *OLFM4* knockout enriched more basal stem/progenitor-like cells, which highly express MYC, in RWPE1 cells.

We further identified genes from whole-genome transcriptome analysis (22,339 genes) with a greater than twofold Log FC change when *OLFM4*-knockout GFP reporter RWPE1 cells were compared with *OLFM4*-wild RWPE1 cells (Log FC OLFM4-KO/OLFM4-W; Supplementary Table S2). This analysis revealed 199 upregulated genes and 1443 downregulated genes. Gene set enrichment analysis (GSEA) revealed an enrichment pattern of gene signatures related to stem cells, such as mammary stem-cell upregulated genes, as well as an embryonic stem-cell signature in *OLFM4*-knockout GFP reporter RWPE1 cells (Fig. 5b). These enriched stem-cell signaling pathway signatures were pinpointed to be for WNT-signaling and APC/MYC-signaling target genes (Fig. 5c and Supplementary Fig. S7)^40^. We verified increased MYC gene expression in *OLFM4*-knockout GFP reporter RWPE1 cells using qRT-PCR, Western blot analysis, and immunofluorescent staining (Supplementary Fig. S7). The MYC target genes signature was found to be enriched in *OLFM4*-knockout GFP reporter RWPE1 cells in data obtained from three different data resources (Fig. 5d and Supplementary Fig. S7). To test function of the *MYC* gene in RWPE1 cells, we used (+)-JQ1, a MYC inhibitor, in both 2D and 3D culture models, and found that (+)-JQ1 substantially inhibited proliferation of *OLFM4*-knockout GFP reporter RWPE1 cells compared with OLFM4-W RWPE1 cells in both types of cultures (Fig. 5e–g). These results provided further evidence that OLFM4 mediates RWPE1 cell proliferative processes through MYC signaling pathways.

### RNA-sequencing data analysis demonstrates potential MYC-related molecular mechanisms of the *OLFM4* gene in RWPE1 stem/progenitor-like cells

**Figure 6:**
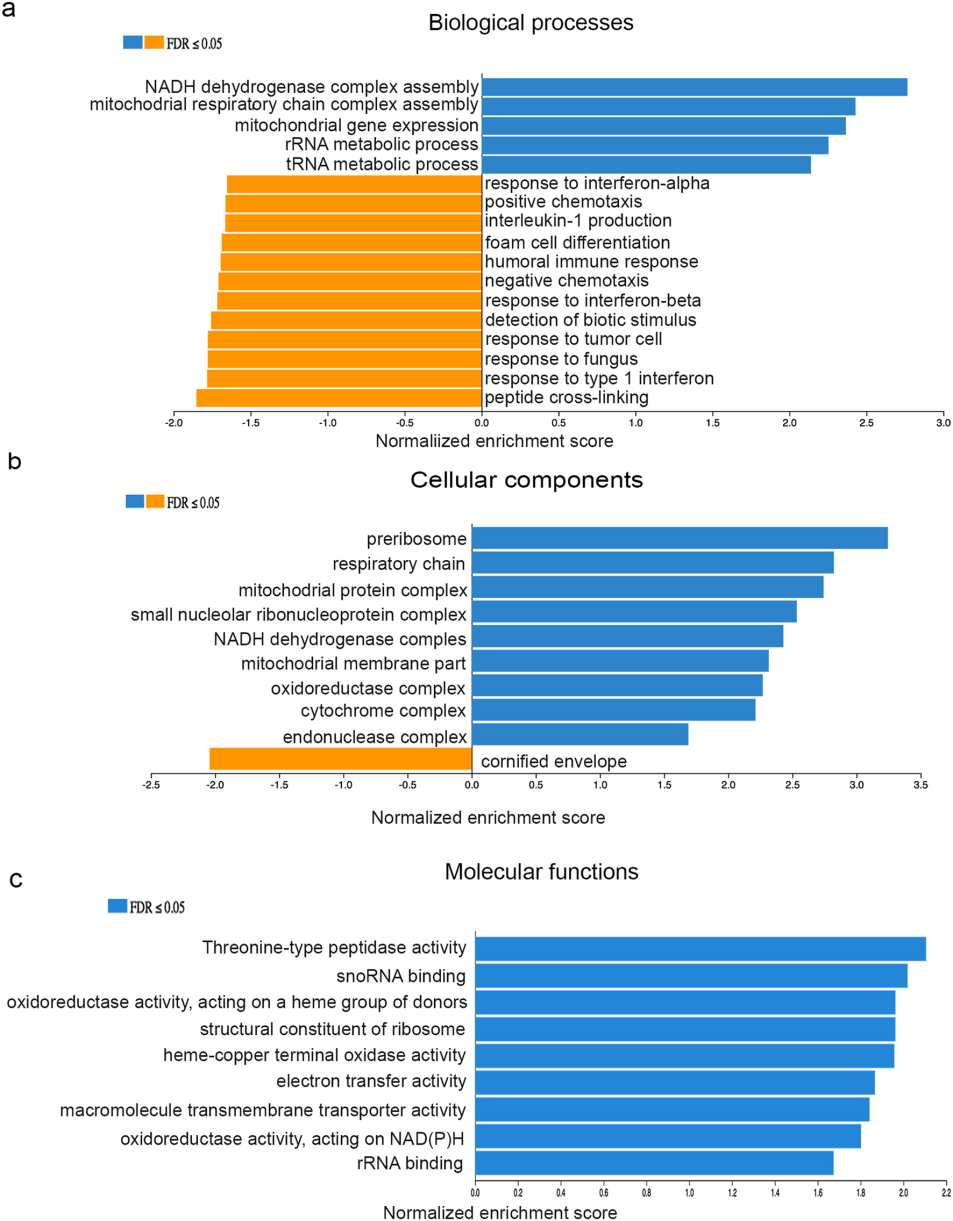
Gene ontology enrichments for *OLFM4*-knockout GFP reporter RWPE1 cells. (**a**) Bar graph presents upregulated (blue; FDR ≤ 0.05) and downregulated (orange; FDR ≤ 0.05) pathways for biological processes using WebGestalt 2019. (**b**) Bar graph presents upregulated (blue; FDR ≤ 0.05) and downregulated (orange; FDR ≤ 0.05) pathways for cellular components. (**c**) Bar graph presents upregulated (blue; FDR ≤ 0.05) pathways for molecular functions.

**Figure 7:**
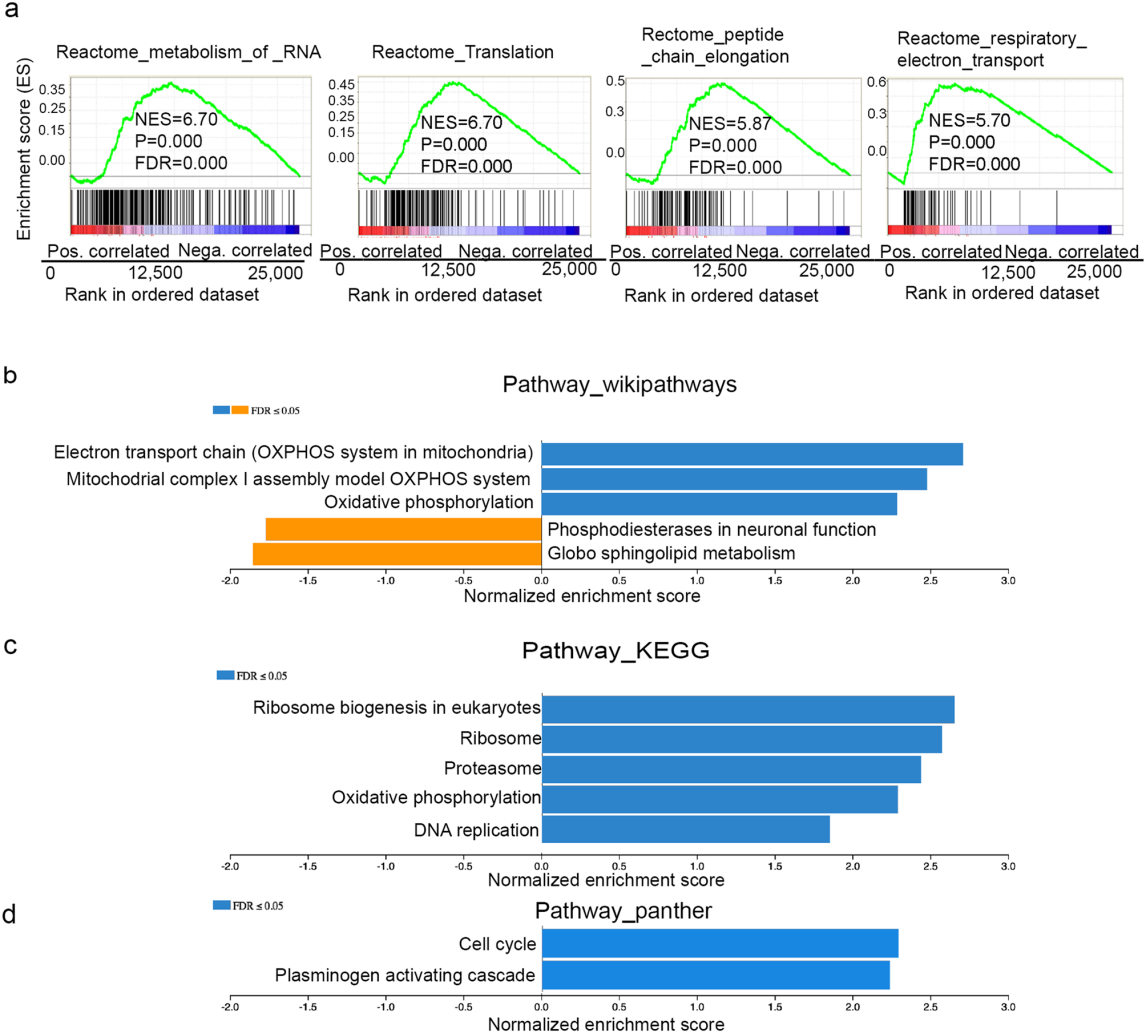
Reactomes and pathways enrichments for *OLFM4*-knockout GFP reporter RWPE1 cells. (**a**) Enrichment of reactomes in *OLFM4*-knockout GFP reporter RWPE1 cells. (**b**) Bar graph presents upregulated (blue; FDR ≤ 0.05) and downregulated (orange; FDR ≤ 0.05) pathways from Wikipathways. (**c**) Bar graph presents upregulated (blue; FDR ≤ 0.05) pathways from Pathway_KEGG. (**d**) Bar graph presents upregulated (blue; FDR ≤ 0.05) pathways from Pathway_Panther.

We further analyzed RNA sequencing data to identify gene ontology enrichments in *OLFM4*-knockout GFP reporter RWPE1 cells compared with *OLFM4*-wild RWPE1 cells. This analysis yielded 5 positively enriched and 12 negatively enriched gene signatures for biological processes (Fig. 6a), 9 positively enriched and 1 negatively enriched gene signature for cellular components (Fig. 6b), and 9 positively enriched gene signatures for molecular functions (Fig. 6c). Further analysis from gene ontology demonstrated significantly enriched reactomes including metabolism of RNA, translation, peptide chain elongation, and respiratory electron transport (Fig. 7a). The pathways analysis revealed 10 significantly enriched pathways, such as Electron transport chain (OXPHOS system in mitochondria) from Wikipathways pathways (Fig. 7b), Ribosome biogenesis in eukaryotes from KEGG pathways (Fig. 7c), and cell cycle from Panther pathways (Fig. 7d). These results suggest that OLFM4 negatively mediates MYC protein functions related to cell proliferation, metabolism, ribosome biogenesis, protein synthesis, and mitochondrial function.

## Discussion

We report for the first time that *OLFM4*-expressing cells represent multiple stem/progenitor-like cell populations and that the *OLFM4* gene plays an important role in cell self-renewal and differentiation. Therefore, the *OLFM4* gene might be useful for lineage tracing of normal prostate stem/progenitor cells during organogenesis and homeostasis of prostate.

Prostate stem/progenitor cells have been identified in the urogenital sinus epithelium, prostatic buds, and solid prostatic tube during prostate organogenesis, as well as in the adult prostate urethra tube epithelium and prostate grands^41,42^. Recently, Henry et al. reported two clusters of stem/progenitor cells in the normal adult prostate epithelium based on their gene expression signature obtained from scRNA sequencing, classifying them as KRT13+ Hillock and SCGB1A1+ Club cells^8^. Because their scRNA sequencing data are publicly available in the GEO database, we performed bioinformatic analysis on those data and found higher OLFM4 expression in cluster 7 (OLFM4+/SCGB3A1+/PSCA+/CD24+) and in cluster 12 (OLFM4+/KRT13+/KRT19+) prostate stem/progenitor cells in normal adult prostate. Due to tissue resource limitations, we used the immortalized human normal adult prostate epithelial cell line, RWPE1, for further studies of *OLFM4*-expressing stem/progenitor-like cell populations. The gene-expression signature of RWPE1 stem/progenitor cells has been shown to conserve most genes in the normal and benign prostate stem/progenitor cell signature^8,9^. Therefore, we used these cells to mimic stem/progenitor cell self-renewal and differentiation in the organogenesis of prostate epithelium.

Our data indicate that OLFM4 is a marker for multiple stem/progenitor-like cell populations within the RWPE1 cell population. In *OLFM4*-expressing stem/progenitor-like RWPE1 cell populations, we identified several stem/progenitor-cell marker genes, such as *LY6D*, *KRT13*, *CD44*, *CD24, ITGA6, and PSCA*. In particular, OLFM4 most frequently co-expressed with *LY6D,* a marker for prostate stem cells that are castration resistant and an origin for prostate cancer^43^. More than half of the OLFM4-expressing RWPE1 cells expressed KRT13, which is highly expressed in Hillock cells localized in the prostatic urethra tube epithelium in what are called prostatic stem cell niches in normal human prostate^8,44^. Almost half of the OLFM4+ RWPE1 cell population also expressed CD24, which is a luminal stem/progenitor cell marker^45^. The luminal progenitor cells have recently been identified as having an underlying role in prostate development, androgen-mediated regeneration of post-castration prostate, and the origin of prostate adenocarcinoma^17,46–48^. Therefore, our identification of multiple OLFM4-expressing stem/progenitor-like cells may contribute to the identification of the cell types of origin in prostatic diseases.

Stem/progenitor cells maintain homeostasis in the adult prostate epithelium and regenerate prostate epithelium after castration, as well as initiate prostate tumorigenesis after targeting by carcinogens^1,38,49,50^. Our finding that *OLFM4*-knockout RWPE1 cells exhibited enhanced proliferation of the CD44+/CD49F+ stem/progenitor-like cell population, shifts stem/progenitor-like cell division to favor symmetric division and differentiated into higher levels PSA expression cells in organoid assays when compared with *OLFM4*-wild RWPE1 cells. *OLFM4*-knockout GFP reporter RWPE1 stem/progenitor-like cells exhibited enhanced self-renewal and disrupted differentiation suggests that OLFM4 plays a role in regulating self-renewal and differentiation of stem/progenitor cells in the normal prostate epithelium and in the initiation of malignant progression. Indeed, *OLFM4*-expressing prostate stem/progenitor cells were immortalized in the RWPE1 cell line, which mimics the initiation of malignant progression of prostate epithelial cells. Therefore, *OLFM4*-expressing stem/progenitor cells may be targets of oncogenic transformation in the progression of prostate cancers.

We explored the molecular mechanisms by which OLFM4 mediates stem/progenitor cell proliferation and differentiation using RNA sequencing, bioinformatics, and whole-genome transcriptome analysis approaches. This analysis revealed that WNT/APC/MYC signaling and MYC target genes were enriched in *OLFM4*-knockout GFP reporter RWPE1 cells. These findings are similar to previously published gene array data that show Wnt-signaling pathway genes were upregulated in colon tissues from *Olfm4*-knockout *Apc*^*Min/*+^ mice^51^. Interestingly, we previously found that *OLFM4* is a Wnt-signaling target gene and negatively regulates the Wnt-signaling pathway through directly binding to frizzled-7 and frizzled-10 in colon-cancer cells^51^. We conclude that knockout of *OLFM4* eliminates a negative control factor of WNT/APC/MYC signaling genes and MYC target genes that regulate multiple biological process and signaling pathways. Therefore, loss of *OLFM4* enhances proliferation and disrupts differentiation of prostate stem/progenitor cells (Supplementary Fig. S8). Taken together, these findings suggest that OLFM4 plays an important role in proliferation and differentiation of prostate stem/progenitor cells through majority mediation of WNT/APC/MYC signaling.

## Methods

### RWPE1 cell line

The immortalized human normal prostate epithelial cell line RWPE1 was purchased from the American Type Culture Collection (ATCC, CRL-11609) and maintained in culture in prostate epithelial cell basal medium (ATCC PCS-440-030) supplemented with the prostate epithelial cell growth kit (ATCC PCS-440-040) in T25 flasks. The RWPE1 cell lines were authenticated and characterized by ATCC, which uses morphology, karyotyping, and PCR-based approaches to confirm the identity of cell lines. All cells were maintained at lower passages.

### Single-cell RNA sequencing for RWPE1 cells

Single RWPE1 cells (passage 2) were prepared and sequenced using the Chromium Single Cell 3′ Reagent Kit V3 (10 X Genomics) following the Single Cell Protocols Guide from the manufacturer. RNA sequencing was performed by the DNA Sequencing and Genomics Core Facility at the National Heart, Lung, and Blood Institute using an Illumina Hi-Seq instrument.

### Single-cell RNA sequencing data analysis

The barcodes, genes, and matrix files resulting from Cell Ranger utility as applied to 10X specific single-cell RNA sequencing for normal adult human prostate with GEO submission number (GSE117403)^8^ were downloaded and analyzed using Seurat R package^52^. The single-cell data-based gene co-regulation network was generated by correlating the dimensionality reduction coordinates. This approach is derived from the Functional Gene mRNA (FGM) profiling method^53^. The FGM profiling method applies principal component analysis (PCA) on the correlation matrix to obtain gene loadings. Finding the significant number of PC’s and correlating genes based on these loadings is the central idea behind FGM profiling method to obtain co-regulation networks. Since scRNA-seq data sets yield in quantified transcriptomes that are noisy, diffusion maps were used instead of PCA for dimensionality reduction (www.helmholtz-muenchen.de/icb/destiny). In our network generation approach, we identify significant diffusion map components (DMC), and correlate the significant DMC’s and retain high correlations (absolute Pearson’s correlation ≥ 0.65) to obtain the network.

### Fluorescent immunohistochemistry and immunocytochemistry

Unstained paraffin section slides of human prostate cancer tissues were purchased from The Cooperative Human Tissue Network (CHTN, Mid-Atlantic Division). Fluorescent immunohistochemistry on paraffin sections or immunocytochemistry on RWPE1 cells was performed as described previously^8^. Images were obtained using the Zeiss 880 Confocal Microscope (inverted). Primary antibodies were used for staining: anti-OLFM4 (OLFM4 (D1E4M) Rabbit mAb, Cell Signaling Technology Inc., #14369); anti-CK13 (clone EPR3671, Abcam, Cat# ab92551); anti-CD44 (NBP1-47386, 8E2F3, Novus Biologicals); chicken anti-CK5 (Biolegend, Cat#905901); anti-SCGB1A1 (clone 394324, Novus Biologicals Cat# MAB4218-SP); anti-FOXA1 (ab 55178, Lot# GR3241742-2, Abcam); and mouse anti-CK8 (MMS-162p-250, 1E8, Covance); anti-AR (N-20, SC-816, Santa Cruz Biotechnology); anti-PSA (5G6, SC-52172, Santa Cruz Biotechnology); anti-P63 ( D9L7L, Rabbit mAb, #39692, Cell Signaling Technology Inc.); anti-CK17/19 (D4G2, Rabbit mAb, #12434, Cell Signaling Technology Inc); anti-CK14 (#MA5-11599, invitrogen); anti-SYN (SY38, ab8049-1, abcam); and anti-MYC (D3N8F, Rabbit mAb, #13987, Cell Signaling Technology Inc).

The images were processed with Fiji 3 software for 2D images and Imais 64-9.21 for 3D images. The combined picture panels were assembled with Adobe Photoshop CC 2017.

### Generation of *OLFM4*-wild or *OLFM4*-knockout GFP reporter RWPE1 cells

To generate OLFM4-wild-GFP reporter cells and OLFM4-knock out-GFP reporter cells, we transfected the CRISPR/Cas 9 OLFM4 activation or knock out plasmids into the RWPE1 *OLFM4*-wild cells (Supplementary Fig. S4). The Double Nickase Plasmid CRISPR/Cas9 knockout plasmid (sc-403599-NIC and sc-403599-NIC-2), the CRISPR/Cas9 activation plasmid (sc-403599-ACT and sc-403599-ACT-2), and the GC-1 HDR-plasmid (h) (sc-403599-HDR) for the human *OLFM4* gene were purchased from Santa Cruz Biotechnology. The plasmid carried gRNA sequencing of the *OLFM4* gene as follows: OLFM4 gRNA sequencing: sc-403599-ACT GC-1 CRISPR Activation Plasmid (h): AATGTTTGGCAGGGGATATC (Intron 4; 18747 bp); sc-403599-ACT-2 GC-1 CRISPR Activation Plasmid (h2): CTTTCAAGGAAGTACCAAGT (Intron 2; 6443 bp); sc-403599-NIC GC-1 Double Nickase Plasmid (h): cgtggacagagtggaacgct (Exon 2; 25593 bp); sc-403599-NIC GC-1 Double Nickase Plasmid (h): cagggaaacagagcactggc (Intron 2; 5844 bp); sc-403599-NIC-2 GC-1 Double Nickase Plasmid (h2): tccagccgcagcttaggcag (Exon 1; 9-205 bp); sc-403599-NIC-2 GC-1 Double Nickase Plasmid (h2): gctggagcccgacctggagc (Intron 1; 1051 bp). The plasmids were transfected into RWPE1 cells using Lipofectamine 2000 reagents (Invitrogen) according to the manufacturer’s instructions. The cells were cultured in the presence of 5 µg/ml puromycin (Invitrogen) to establish stable cell lines.

### Cell sorting and FACS analysis

*OLFM4*-wild GFP reporter and *OLFM4*-knockout GFP reporter RWPE1 cells were grown in prostate epithelium growth medium (ATCC) to 80% confluence in T25 flasks. Cells were harvested by trypsinization, removed from flasks, and centrifuged at 1000 rpm for 5 min at room temperature.

For cell sorting, 1–2 × 10^7^ cells/ml were washed once with medium, then resuspended in 1 ml medium. Anti-GFP Alexa Fluor 488-conjugated antibody (1:200; Invitrogen) was added to the cell suspension and incubated on ice for 30 min. After washing and filtering with a cell strainer (100 µm filter, BD Falcon), FACS-based cell sorting was performed with BD Aria (BD Biosciences).

For FACS analysis, 1–2 × 10^6^ cells/ml were resuspended in 1 ml PBS and stained with the following antibodies: anti-GFP Alexa Fluor 488-conjugated antibody (1:200; Invitrogen) anti-CD49F PE-conjugated antibody (1:200, eBioscience); anti-CD44 PE-conjugated antibody (1:200, eBioscience); anti-CD26 PE-conjugated antibody (1:100, eBioscience); or anti-CD24 PE-conjugated antibody (1:100, eBioscience) for 1 h at room temperature, mixing with rotation. The cells were then washed with PBS once and resuspended in 0.5 ml PBS, then analyzed by flow cytometry (BD FACSCalibur, BD Biosciences).

### Prostate sphere-formation assay

Prostate sphere-formation assays were performed following a previously described protocol^54^. Briefly, 1 × 10^4^ *OLFM4*-wild or *OLFM4*-knockout GFP reporter RWPE1 cells were suspended in 50 µl growth medium and mixed with 50 µl Matrigel, then cultured in 12-well plates for up to 14 days. For cells treated with 100 nM DHT (Sigma-Aldrich, #A8380) or 0.1 to 1 μM (+)-JQ1 (Sigma-Aldrich, #SML1524), the treatment medium was replaced with fresh medium containing the treatment reagent every 2 days. Dimethyl sulfoxide (DMSO) was used as a vehicle control for all treatment reagents. Images of spheres were captured with an AX10 cam 503 mono or GFP AX10 Cam 105 Color with a ZEISS microscope (AX 10) and ZEISS software for different timepoints, and GFP-positive colonies larger than 50 µm in diameter were counted.

### Organoid culture

Organoid culture was performed following the protocol published previously by Drost et al.^1^ Briefly, 4 × 10^5^ *OLFM4*-wild or *OLFM4*-knockout GFP reporter RWPE1 cells were placed with 40 µl Matrigel in the center of each well in a 24-well plate. Human organoid culture medium was prepared following the protocol and 0.5 ml was added to each well. Organoid growth was traced from day 1 to day 12 by taking pictures of GFP-expressing single cells using a ZEISS AXIO microscope with either a GFP filter or using a light field. Organoid images were processed with Adobe Photoshop software. For immunofluorescence staining, organoids that had been cultured for 12 days were fixed with 10% formalin solution (Sigma-Aldrich, # HT5011) in PBS at room temperature for 1 h, then changed into 70% ethanol overnight. After paraffin embedding, 5-µm sections of organoids were cut, and paraffin section slides used for fluorescent immunohistochemistry.

### Colony-formation assay and (+)-JQ1 treatment

RWPE1 cells were plated at 1 × 10^4^ cells/well in precoated six-well plates and grown overnight in prostate epithelial cell basal medium supplemented with the prostate epithelial cell growth kit. The next day, the medium was changed to treatment medium containing DMSO (vehicle control) or 1–10 µM (+)-JQ1 and cultured for 7 days. The treatment medium was replaced with fresh medium containing the treatment reagent every 2 days. The cells were then stained with the Diff-Quik Stain Set (Data Behring Inc.) and photographed. The cell images were photographed under light (AX10 cam 503 mono) or fluorescent (AX10 Cam 105 Color) conditions with a ZEISS microscope (AX 10) and ZEISS software for different timepoints. All images were processed with Adobe Photoshop for presentation.

### Bulk-cell RNA sequencing and data analysis

Total RNA was purified from GFP-sorted bulk RWPE1 cells using RNeasy plus Mini kits. RNA sequencing was performed by the DNA Sequencing and Genomics Core Facility at the National Heart, Lung, and Blood Institute using an Illumina Hi-Seq instrument. Briefly, sequencing libraries were constructed from 100–500 ng of total RNA using the Illumina TruSeq Stranded Total RNA kit with Ribo-Zero following the manufacturer’s instructions. The fragment size of the RNAseq libraries was verified using an Agilent 2100 Bioanalyzer and the concentrations determined using a Qubit instrument (LifeTech). The libraries were loaded onto the Illumina HiSeq 3000 for 2 × 75 bp paired-end read sequencing. The FASTQ files were generated using Illumina bcl2fastq conversion software (https://support.illumina.com/sequencing/sequencing_software/bcl2fastq-conversion-software.html) for further whole-genome transcriptome analysis.

Quality control of FASTQ files was assessed using the FastQC toolkit (v0.11.5) using default parameters. The paired-end reads were aligned against the human reference genome (GENCODE GRCh38) using HISAT2 (v2.0.5) alignment^55^. Gene-level read counts were produced by featureCounts (v1.5.2)^56^ using only uniquely mapped, paired-end, reversely stranded reads. Differential-expression analysis at the gene level was conducted using limma-voom open-source R packages^57,58^. TMM (Trimmed Mean of M values) normalization was conducted, and normalized factors were estimated for each sample^59^. The lmFit package in limma-voom was used to fit linear models for each gene to calculate log2-fold changes and *p* values using the normalized factors as weights in the model. To account for multiple testing, the false discovery rate (FDR) via the Benjamani–Hochberg algorithm^60^ was calculated^61^.

We characterized differentially expressed genes (DEGs) with respect to both gene ontology (GO) and pathway enrichment to assess the functional association of the DEGs. Gene set enrichment for GO^62^ including biological process, cellular component, and molecular function analysis, Kyoto Encyclopedia of Genes and Genomes (KEGG) analysis^63^, Panther Database^64^, and Wikipathway^65^ were performed using WebGestalt^66^ using *p* < 0.05 as the cut-off criterion. Gene set enrichment analysis (GSEA) was conducted to identify the significantly upregulated and downregulated pathways between high and low DEGs using FDR < 0.05 as the cut-off criterion.

### Statistical analysis

Student’s t-test was used to analyze differences between groups. *p *values < 0.05 were considered statistically significant.

## Supplementary information

Supplementary Information 1.

Supplementary Information 2.

Supplementary Information 3.

## Data Availability

RNA sequencing data that support the findings of this study have been deposited in the GEO database, with the accession code GSE126162 (https://www.ncbi.nlm.nih.gov/geo/query/acc.cgi?acc=GSE126162).
