# Supplementary material for: Olfactomedin 4 mediation of prostate stem/progenitor-like cell proliferation and differentiation via MYC
Source: Sci Rep. 2020 Dec 14;10:21924. doi: 10.1038/s41598-020-78774-5 (PMC7736579; doi:10.1038/s41598-020-78774-5)
Supplement: Supplementary file 1 — Supplementary Information 1. [file 41598_2020_78774_MOESM1_ESM.docx]

**Supplementary Information (8 Figures and Methods)**

**Olfactomedin 4 mediation of prostate stem/progenitor-like cell proliferation and differentiation via MYC**

Hongzhen Li^1^, Vijender Chaitankar^2^, Jianqiong Zhu^1^, Kyung Chin^1^, Wenli Liu^1^, Mehdi Pirooznia^2^, and Griffin P. Rodgers^1,*^

^1^Molecular and Clinical Hematology Branch, National Heart, Lung, and Blood Institute, National Institutes of Health, Bethesda, MD 20892, USA

^2^Bioinformatics and Systems Biology Core, National Heart, Lung, and Blood Institute, National Institutes of Health, Bethesda, MD 20892, USA

*Corresponding author: Griffin P. Rodgers, MD, Molecular and Clinical Hematology Branch, National Heart, Lung, and Blood Institute, National Institutes of Health, Bldg. 10, Room 9N119, 9000 Rockville Pike, Bethesda, MD 20892. Tel: 301 402 2418. Fax: 301 480 1940. E-mail: gr5n@nih.gov


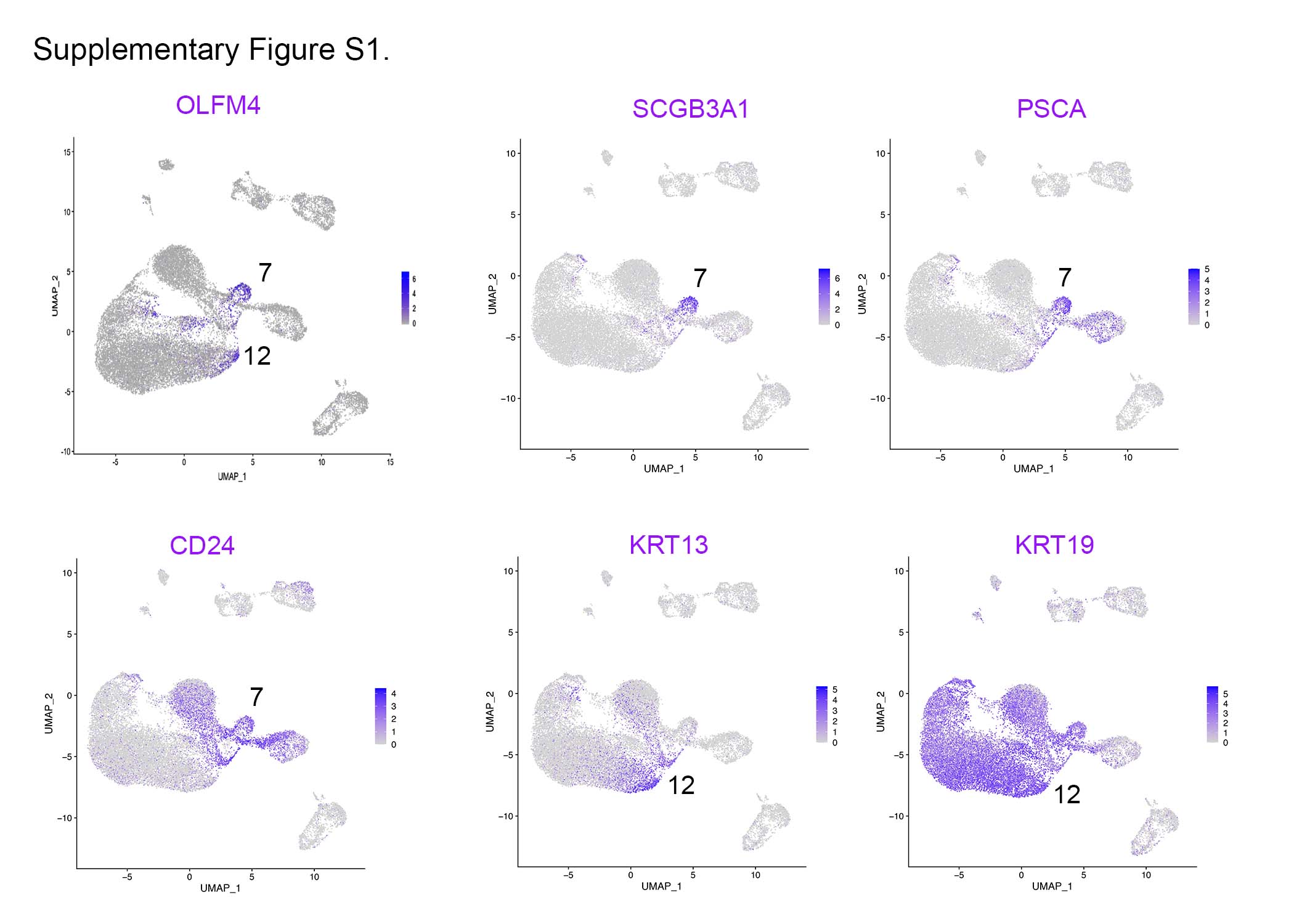


**Supplementary Figure S1. OLFM4 co-expression with stem/progenitor cell markers in normal human adult prostate epithelium.** Uniform Manifold Approximation and Projection (UMAP) plots of integrated data from single-cell RNA sequencing of the GSE117403 dataset. OLFM4 is co-expressed with SCGB3A1, PSCA, and CD24 in cluster 7 and with KRT13 and KRT19 in cluster 12.


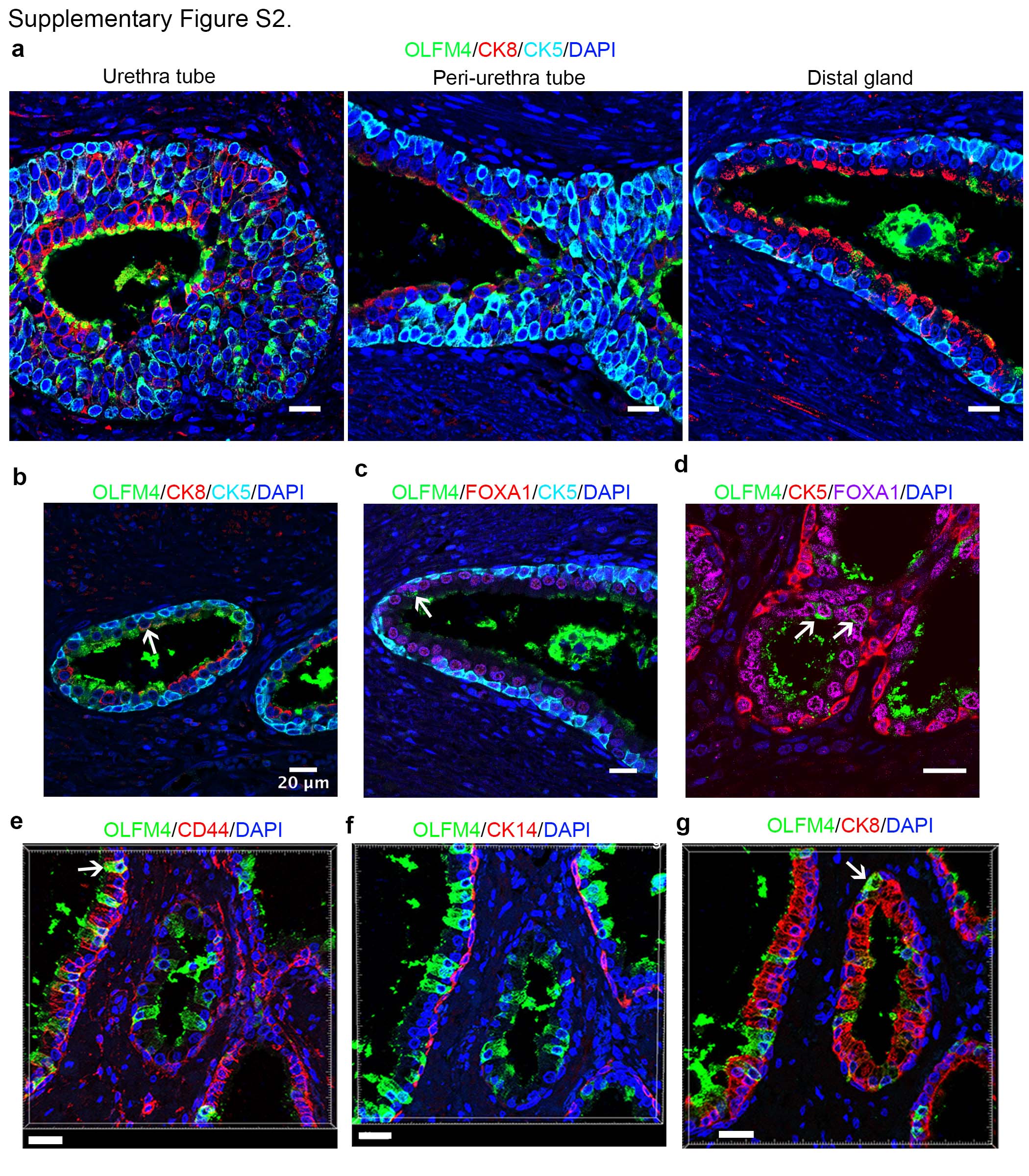


**Supplementary Figure S2. Identification of *OLFM4*-expressing cells in normal human adult prostate epithelium.** (**a**) Representative triple-color immunofluorescent staining of normal prostatic urethra tube (left panel), peri-urethra tube (middle panel), and distal gland (right panel) epithelium. Scale bar: 20 μm. (**b–d**) Representative triple-color immunofluorescent staining of normal prostatic acini epithelium. Arrows indicate OLFM4+/CK8+/FOXA1+ cells. Scale bar: 20 μm. (**e–g**) Representative double-color immunofluorescent staining of normal prostatic acini epithelium. Arrows indicate OLFM4+/CD44+ (**e**) and OLFM4+/CK8+ (**g**) cells. Scale bar: 20 μm.


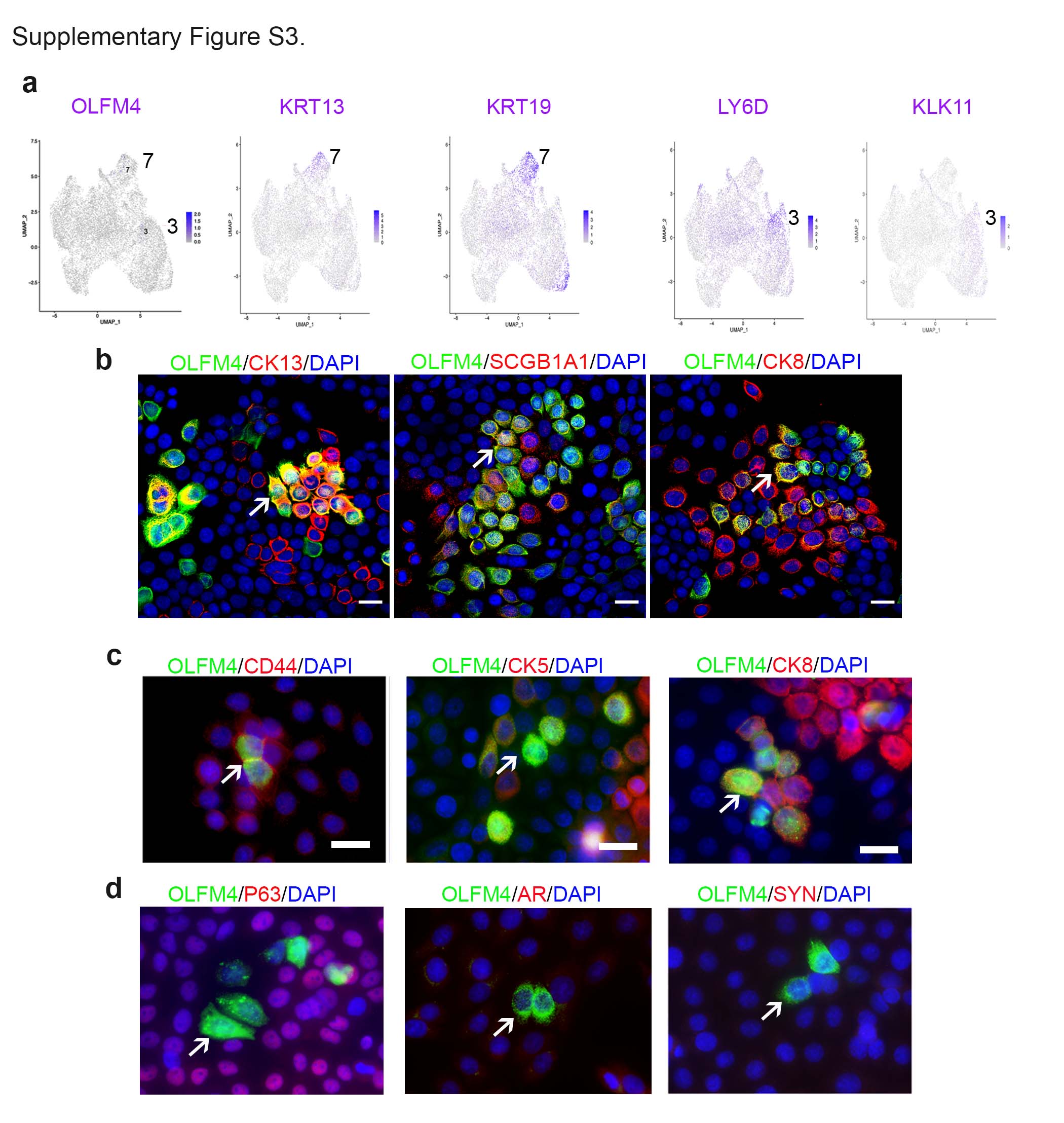


**Supplementary Figure S3. Identification of OLFM4-expressing cells RWPE1 cells.** (**a**) Uniform Manifold Approximation and Projection (UMAP) plots of integrated data from single-cell RNA sequencing of RWPE1 cells. OLFM4 is co-expressed with KRT13 and KRT19 in cluster 7 and with LY6D and KLK11 in cluster 3. (**b**) Representative double-color immunofluorescent staining of RWPE1 cells. OLFM4 (green) cell marker (red); DAPI (blue). Arrows indicate double-positive expression cells. Scale bar: 20 μm. (**c**) Representative double-color immunofluorescent staining of RWPE1 cells. OLFM4 (green); cell marker (red); DAPI (blue). Arrows indicate double-positive expression cells. Scale bar: 50 μm. (**d**) Representative double-color immunofluorescent staining of RWPE1 cells grown in the presence of 100 nM DHT. OLFM4 (green); cell marker (red; P63 [4A4, Cat# ab735, Abcam], androgen receptor [PG-21; Cat#06680, Lot# 2275569, Millipore], and synaptophysin [ab8049-1, SY38, Lot# 447758, Abcam]); DAPI (blue). Scale bar: 50 μm. AR, Androgen receptor; SYN, Synaptophysin. Arrows indicate OLFM4+ cells.


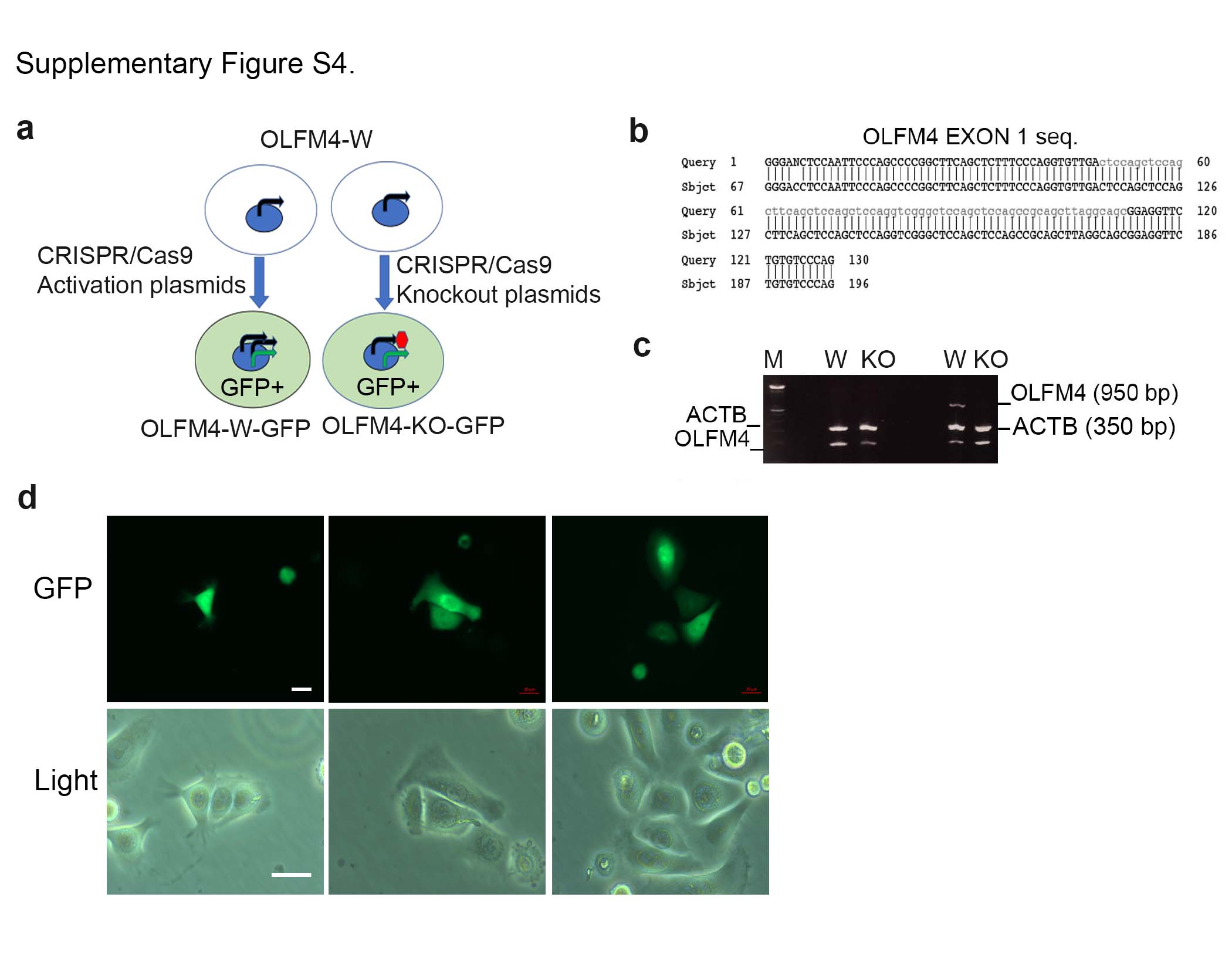


**Supplementary Figure S4. Generation and identification of the GFP reporter RWPE1 cell lines.** (**a**) Diagram outlining approach used to establish *OLFM4*-wild GFP reporter (OLFM4-W-GFP) RWPE1 cells and *OLFM4*-knockout GFP reporter (OLFM4-KO-GFP) RWPE1 cells with *OLFM4* CRISPR/Cas 9 activation and knockout plasmids. Black bend arrows indicate *OLFM4* expression. Green bend arrows indicate GFP protein expression. Red hexagon indicates OLFM4-knock out. (**b**) Genomic sequence of *OLFM4* exon 1 showing the knockout region. (**c**) Semi-quantitative RT-PCR showing OLFM4 RNA expression with two pairs of probes. PCR product sizes: 220 bp and 950 bp. β-actin (ACTB) was used as an internal control. W, *OLFM4*-wild cells; KO, *OLFM4*-knockout cells. (**d**) Representative GFP- and light-field images of *OLFM4*-W-GFP RWPE1 cells after 7 days in 2D culture. Scale bar: 10 μm.

**
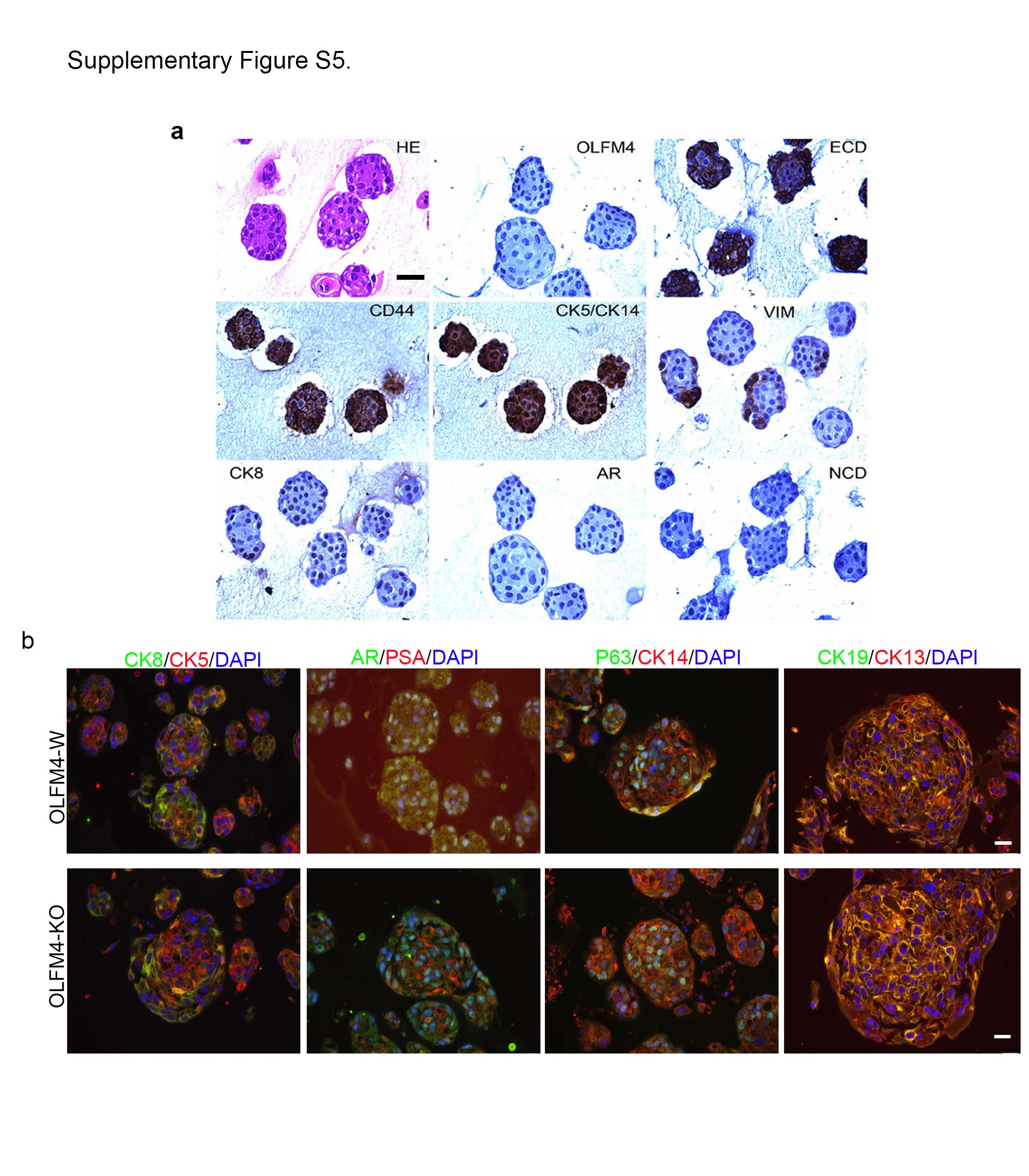
Supplementary Figure S5. Identification of prostate spheres and** organoids with IHC and IFC (**a**) Representative images of staining of prostate spheres formed after 14 days in Matrigel culture in the presence of DHT (100 nM) for HE, OLFM4, E-cadherin (ECD), CD44, CK5/CK14, CK8, vimentin (VIM), AR, and N-cadherin (NCD). HE, hematoxylin-eosin staining. Scale bar: 100 μm. (**b**) Representative double-color immunofluorescent staining of small organoids at 12 days in culture. DAPI (blue) was used for nuclei staining. Scale bars: 20 μm.


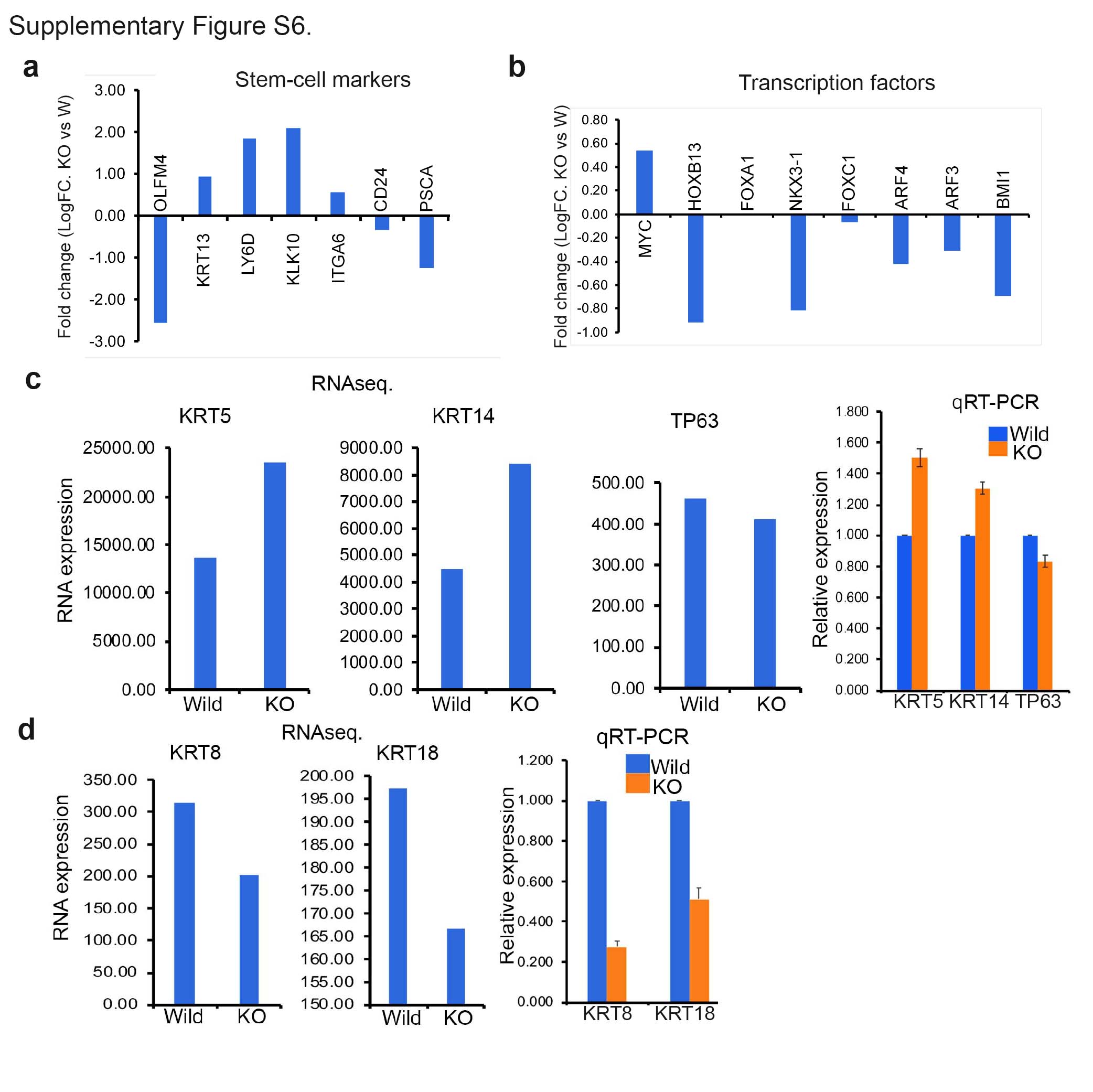


**Supplementary Figure S6. Altered expression of genes in *OLFM4*-knockout GFP reporter RWPE1 cells from bulk-cell RNA sequencing.** (**a**) Representative fold changes (logFC, KO vs. Wild [W]) in stem-cell marker genes. (**b**) Representative fold changes (logFC, KO vs. Wild [W]) in transcription factor genes. (**c**) Representative RNA expression and qRT-PCR for KRT5, KRT14, and TP63 in RWPE1 cells. (**d**) Representative RNA expression and qRT-PCR for KRT8 and KRT18 in RWPE1 cells. qRT-PCR data are presented as mean ± SD (n=3). Wild, *OLFM4*-wild cells; KO, *OLFM4*-knockout cells.


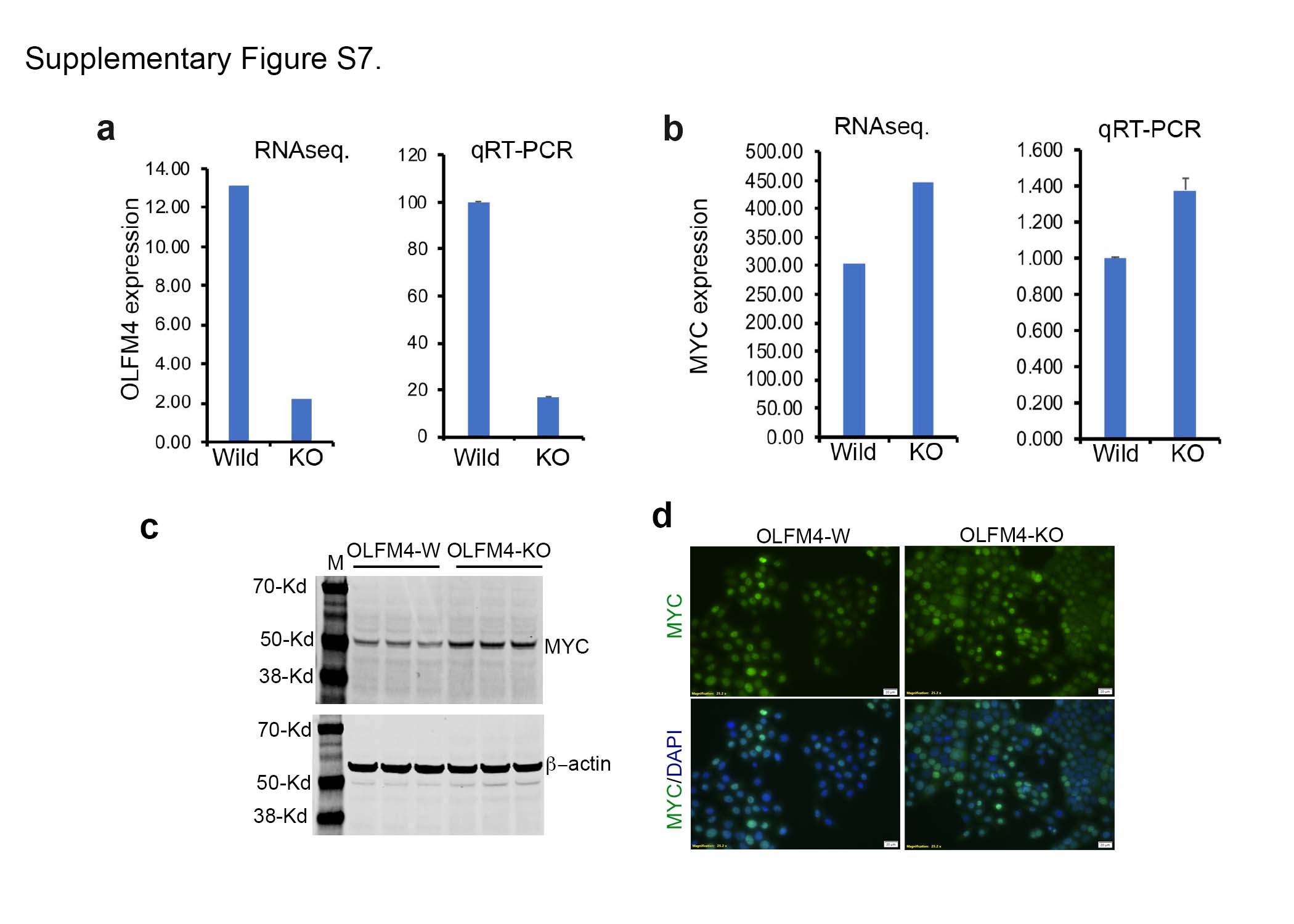


**Supplementary Figure S7. Altered expression of genes in *OLFM4*-knockout GFP reporter RWPE1 cells from bulk-cell RNA sequencing.** (**a**, **b**) Representative RNA expression and qRT-PCR for *OLFM4* (**a**) and *MYC* (**b**) genes in RWPE1 cells. qRT-PCR data are presented as mean ± SD (n=3). (**c**) Representative Western blot of MYC protein expression in *OLFM4*-wild (W) GFP reporter and *OLFM4*-knockout (KO) GFP reporter RWPE1 cells. β-actin was used as a loading control. M, molecular-weight markers. (**d**) Representative immunofluorescent staining of *OLFM4*-W GFP reporter and *OLFM4*-KO GFP reporter RWPE1 cells. MYC (green) anti-MYC (D3N8F, Rabbit mAb, #13987, Cell Signaling Technology Inc); DAPI (blue) was used for nuclei staining. Scale bars: 20 μm.


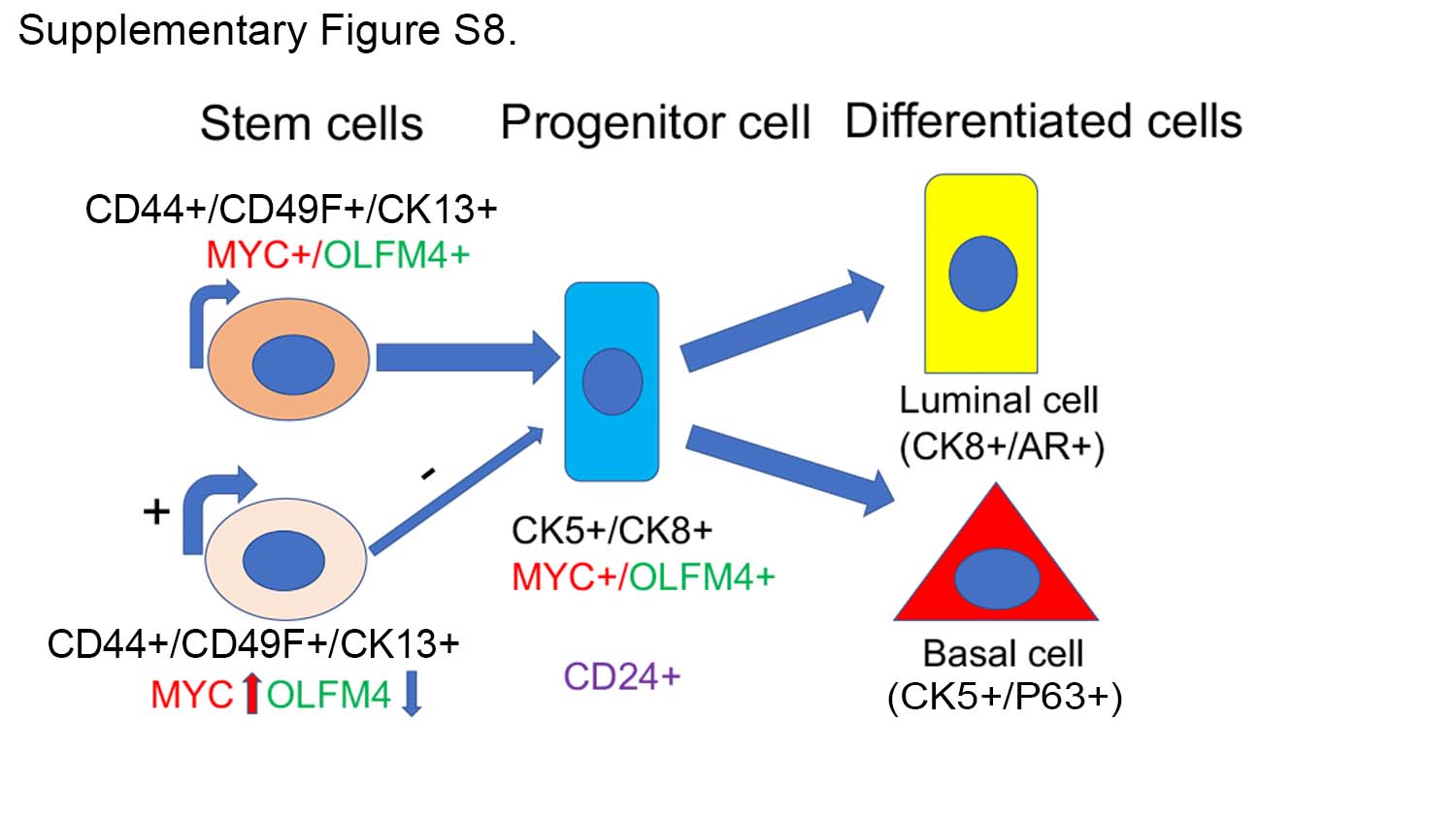


**Supplementary Figure S8. Illustration of prostate stem/progenitor cell self-renewal and differentiation under conditions of *OLFM4* expression or loss**. *OLFM4*-expressing stem cells (OLFM4+/CK13+/CD44+/CD49F+; top left) do self-renew, but preferentially differentiate into intermediate progenitor cells (OLFM4+/CD24+/CK5+/CK8+; middle) and then further terminally differentiate into CK8+/AR+ luminal cells and CK5+/P63+ basal cells (right). When *OLFM4* was downregulated in OLFM4+/CK13+/CD44+/CD49F+ stem cells, *MYC* gene function was upregulated (bottom left), shifting stem cells towards self-renewal and reducing differentiation. +, enhance; -, reduce.

**Supplementary Methods**

**Immunohistochemical staining.** Immunohistochemical staining of prostate spheres from 3D cultures was performed with the following primary antibodies: anti-OLFM4 (OLFM4 (D1E4M) Rabbit mAb, #14369, Cell Signaling Technology Inc.); anti-AR (D6F11, Cat# 5153, Cell Signaling Technology Inc.); 34βE12 (M0630, mouse anti-human cytokeratin HMW; Dako); anti-CK8 (MMS-162p-250, 1E8, Covance); anti-CD44 (NBP1-47386, 8E2F3, Novus Biologicals); anti-E-cadherin (24E10, Cat# 3195, Cell Signaling Technology Inc.); anti-vimentin (D21H3, Cat# 574, Cell Signaling Technology Inc.); and anti-N-cadherin (D4R1H, Cat# 13116, Cell Signaling Technology Inc.). Secondary antibodies, Super Sensitive MultiLink, and Super Sensitive Label were purchased from BioGenex. Dark brown color was developed with chromagen (BioGenex), and slides were counterstained with hematoxylin (Sigma-Aldrich).

All images were acquired using an Olympus BX51 microscope and Qimaging Camera with Q Capture pro software. Images were acquired using the x40 or x60 Uplan Apo objective (1.42 oil), then imported into Adobe Photoshop for presentation.

**Quantitative real‐time RT‐PCR**

Quantitative real‐time RT‐PCR (qRT‐PCR) was conducted as previously described ^2^ .Briefly, total RNA was extracted from prostate cells using RNeasy plus Mini kits (Qiagen). Total RNA (2 μg) was then reverse‐transcribed using the SuperScript III First‐Strand Synthesis System (ThermoFisher Scientific). TaqMan PCR primers and probes for OLFM4 (Hs00197437_m1), c-MYC (Hs01570247-m1), KRT8 (Hs01595539-g1), KRT18 (Hs02827483_g1), KRT14 (Hs00265033_m1), KRT5 (Hs00361185_m1), TP63 (Hs00978343_m1) and the internal control ACTB (Hs01060665_g1) were purchased from Applied Biosystems (ThermoFisher Scientific). qRT‐PCR was performed with QuantStudio 6 Flex (Applied Biosystems) using the following thermocycler protocol: 94 °C for 10 min, followed by 40 cycles at 94 °C for 10 sec, 60 °C for 30 sec. Relative expression was calculated by a comparative CT method using the formula 2^−ΔΔCT^.

### Western blot analysis. Western blots were performed as previously described ^1^. Briefly, total proteins (25 μg) from whole‐cell extracts were separated electrophoretically using NuPAGE 4–12% Bis‐Tris gels (Invitrogen), transferred to polyvinylidene difluoride membrane, and hybridized with anti-MYC (c-MYC 9E10 #SC-40, Santa Cruz Biotechnology) or anti-β-actin (β-actin C4, #SC-47778, Santa Cruz Biotechnology) antibodies overnight at 4 °C. Blots were then incubated with the goat anti-mouse secondary antibody IRDye^®^ 800CW (#926-32210, LI-COR). The blots were developed using Image Studio Ver 5.2 with the ODYSSEY CLx machine (LI-COR) and were processed with Adobe Photoshop.

1 Li, H. *et al.* Olfactomedin 4 downregulation is associated with tumor initiation, growth and progression in human prostate cancer. *Int J Cancer* **146**, 1346-1358 (2020).
